# Extracellular Vesicles From LPS-Treated Macrophages Aggravate Smooth Muscle Cell Calcification by Propagating Inflammation and Oxidative Stress

**DOI:** 10.3389/fcell.2022.823450

**Published:** 2022-03-09

**Authors:** Linda Yaker, Abdellah Tebani, Céline Lesueur, Chloé Dias, Vincent Jung, Soumeya Bekri, Ida Chiara Guerrera, Saïd Kamel, Jérôme Ausseil, Agnès Boullier

**Affiliations:** ^1^ MP3CV-UR7517, CURS-University of Picardie Jules Verne, Amiens, France; ^2^ INSERM U1245, CHU Rouen, Normandie University, UNIROUEN, Rouen, France; ^3^ Department of Metabolic Biochemistry, Rouen University Hospital, Rouen, France; ^4^ Infinity, INSERM UMR1291, CNRS UMR5051, University of Toulouse III, Toulouse, France; ^5^ INSERM US24/CNRS UAR3633, Proteomic Platform Necker, University of Paris—Federative Research Structure Necker, Paris, France; ^6^ Laboratory of Biochemistry, CHU Amiens-Picardie, Amiens, France; ^7^ Service de Biochimie, Institut Fédératif de Biologie, CHU Toulouse, Toulouse, France

**Keywords:** extracellular vesicles, vascular calcifcation, inflammation, oxidative stress, macrophages

## Abstract

**Background:** Vascular calcification (VC) is a cardiovascular complication associated with a high mortality rate among patients with diseases such as atherosclerosis and chronic kidney disease. During VC, vascular smooth muscle cells (VSMCs) undergo an osteogenic switch and secrete a heterogeneous population of extracellular vesicles (EVs). Recent studies have shown involvement of EVs in the inflammation and oxidative stress observed in VC. We aimed to decipher the role and mechanism of action of macrophage-derived EVs in the propagation of inflammation and oxidative stress on VSMCs during VC.

**Methods:** The macrophage murine cell line RAW 264.7 treated with lipopolysaccharide (LPS-EK) was used as a cellular model for inflammatory and oxidative stress. EVs secreted by these macrophages were collected by ultracentrifugation and characterized by transmission electron microscopy, cryo-electron microscopy, nanoparticle tracking analysis, and the analysis of acetylcholinesterase activity, as well as that of CD9 and CD81 protein expression by western blotting. These EVs were added to a murine VSMC cell line (MOVAS-1) under calcifying conditions (4 mM Pi—7 or 14 days) and calcification assessed by the o-cresolphthalein calcium assay. EV protein content was analyzed in a proteomic study and EV cytokine content assessed using an MSD multiplex immunoassay.

**Results:** LPS-EK significantly decreased macrophage EV biogenesis. A 24-h treatment of VSMCs with these EVs induced both inflammatory and oxidative responses. LPS-EK-treated macrophage-derived EVs were enriched for pro-inflammatory cytokines and CAD, PAI-1, and Saa3 proteins, three molecules involved in inflammation, oxidative stress, and VC. Under calcifying conditions, these EVs significantly increase the calcification of VSMCs by increasing osteogenic markers and decreasing contractile marker expression.

**Conclusion:** Our results show that EVs derived from LPS-EK–treated-macrophages are able to induce pro-inflammatory and pro-oxidative responses in surrounding cells, such as VSMCs, thus aggravating the VC process.

## 1 Introduction

Vascular calcification (VC) is a cardiovascular complication found among patients with diseases such as diabetes, atherosclerosis, and chronic kidney disease (CKD) (Lee et al., 2020). VC is an active process (Demer and Tintut, 2008; Voelkl et al., 2019), characterized by an imbalance of calcium/phosphate homeostasis and hydroxyapatite mineral deposition, both in the intimal and medial layers of the artery (Demer and Tintut, 2008; Drüeke and Massy, 2011; Lee et al., 2020). Vascular smooth muscle cells (VSMCs), which represent the most abundant cell type in vessels, play a pivotal role in the initiation and development of VC (Jaminon et al., 2019). VSMCs undergo a phenotypic switch, with modification of osteogenic, contractile, and synthetic marker expression (Durham et al., 2018). Other cell types may be involved in the induction of the VC process, such as macrophages (Li Y. et al., 2020) and endothelial cells. Indeed, macrophages play an important role in the progression of VC by secreting inflammatory factors and inducing oxidative stress (Hénaut et al., 2019). As a consequence, macrophage-induced inflammation can reduce the production of VC inhibitors, such as fetuin-A (Moe and Chen, 2005), a protein that can bind excess mineral and increase their plasma solubility (Komaba and Fukagawa, 2009). Furthermore, an increase in reactive oxygen species (ROS) production is involved in VSMC osteochondrogenic trans-differentiation during the VC process (Tóth et al., 2020; Hu et al., 2021). Recent studies have highlighted the role of extracellular vesicles (EVs) in VC (Hodroge et al., 2017; Mansour et al., 2020; Yaker et al., 2020; Qin et al., 2021). These membrane-bound vesicles, secreted by prokaryotic and eukaryotic cells (Woith et al., 2019), can be of various origins, depending on their mode of biogenesis. For example, exosomes (50–150 nm) originate from endosomes and microvesicles (50–500 nm) generated by budding of the plasma membrane and apoptotic bodies (van Niel et al., 2018). Several studies have showed macrophage-derived EVs to promote VC (New et al., 2013; Chen et al., 2016; Kawakami et al., 2020). Analysis of their content identified a subset of molecules involved in inflammation and oxidative stress, such as pro-inflammatory cytokines (Fitzgerald et al., 2018; Aiello et al., 2020) and oxidant machinery proteins (Bodega et al., 2019). New *et al.* showed that macrophages can release calcifying EVs enriched for S100A9, a calcium-binding protein involved in mineralization (New et al., 2013). Furthermore, Kawakami *et al.* recently showed that calcifying EVs released by macrophages contribute to the formation of microcalcification (Kawakami et al., 2020). In addition, Chen *et al.* demonstrated that the cytokine HMGB1 can induce the secretion of macrophage-derived EVs involved in ectopic mineralization (Chen et al., 2016). Here, we aimed to investigate the role of macrophage-derived EVs in the propagation of inflammation and oxidative stress during the VC process.

## 2 Materials and Methods

### 2.1 Cell Culture, Molecular, and Biochemical Reagents

Fetal bovine serum (FBS) and glutamine were purchased from Eurobio^®^ (Les Ulis, France). Lipopolysaccharide from *Escherichia coli K12* (LPS-EK) was obtained from InvivoGen^®^ (San Diego, California, United States) and inorganic phosphate (Pi) from Merck^®^ (Darmstadt, Germany). Exosome-free FBS, TRIzol^™^ Reagent, RNase/DNase-free water, High-Capacity RNA-to-cDNA^™^ kits, BCA^™^ Protein Assay kits, and dihydroethidium (DHE) were purchased from Thermo Fisher Scientific^®^ (Waltham, Massachusetts, United States). Takyon^™^ was obtained from Eurogentec^®^ (Liège, Belgium). 2′, 7′-dichlorofluorescein diacetate (DCFH-DA) was purchased from Molecular Probes^®^ (Eugene, Oregon, United States). All other molecular and biochemical reagents were obtained from Sigma-Aldrich^®^ (Saint-Louis, Missouri, United States).

### 2.2 Culture and Treatment of Cells

#### 2.2.1 Murine Macrophage Culture

Murine macrophages (RAW 264.7 ATCC^®^ TIB-71^™^, Manassas, Virginia, United States) were maintained in DMEM 6546 medium supplemented with 10% FBS, 4 mM glutamine, 100 UI/ml penicillin, and 100 µg/ml streptomycin at 37°C in a 5% CO_2_ humidified atmosphere. We first assessed RAW cell viability in the presence of 0.1 and 1 µg/ml LPS-EK, concentrations that are generally used to activated RAW macrophages (Pi et al., 2014). A 24-h treatment with LPS-EK induced 25% cell cytotoxicity (data not shown). RAW cells are known to be particularly sensitive to LPS, which could explain the cell growth inhibition observed in our experiment (Raschke et al., 1978). We then tested the treatment of RAW cells for only 6 h with LPS-EK and detected no cell cytotoxicity (Supplementary Figure S1). We thus used these experimental conditions for all further experiments.

#### 2.2.1 Murine Aortic Vascular Smooth Muscle Cell Culture

Murine aortic VSMCs (MOVAS-1 ATCC^®^ CRL-2797^™^, Manassas, Virginia, United States) were maintained in DMEM 6546 medium supplemented with 10% FBS, 4 mM glutamine, 100 UI/ml penicillin, 100 µg/ml streptomycin, and 200 µg/ml geneticin^®^ (G418) at 37°C in a 5% CO_2_ humidified atmosphere. To induce VSMC calcification, cells were treated in DMEM 6546 containing 1% FBS with 4 mM Pi for 14 days. The media was changed twice a week. For each experiment, VSMCs were treated with macrophage-derived EVs isolated from the same volume of cell-culture medium.

### 2.3 Extracellular Vesicle Preparation and Characterization

#### 2.3.1 Isolation of Extracellular Vesicles

Macrophages were seeded in 10-cm petri plates at a density of 30,000 cells/cm^2^ and cultured in DMEM 6546 medium supplemented with 10% FBS. After 48 h, cells were washed with PBS then treated with various concentrations of LPS-EK in DMEM 6546 medium supplemented with 10% exosome-free FBS for 6 h. Cell debris was removed from cell-culture supernatants by centrifugation at 800 × g for 5 min at 4°C.

EVs were isolated from cell-culture supernatants of untreated (EV-CT) or LPS-EK treated (EV-LPS) macrophages by sequential centrifugation, as described previously by Chen et al. (2008). Briefly, supernatants were first ultracentrifuged at 100,000 × g for 50 min at 4°C. The pellet was then resuspended in cold Dulbecco’s phosphate buffered saline (D-PBS) and centrifuged at 100,000 × g for another 50 min at 4°C. The final pellet was resuspended in D-PBS or RIPA buffer and the protein concentration determined using the BCA^™^ Protein Assay kit. EV samples were stored at −80°C for future analysis.

#### 2.3.2 Characterization of Extracellular Vesicles

##### 2.3.2.1 Transmission Electron Microscopy

EVs were isolated as described previously and resuspended in 50 µL Tris base buffer (100 mM, pH 7.4). EV samples were prepared for TEM using the conventional negative staining procedure. Briefly, 10 µL EV samples were absorbed for 2 min on formvar-carbon-coated copper grids preliminarily ionized using the PELCO easiGlow™ Glow Discharge Cleaning System (Ted Pella Inc., Redding, California, United States). Preparations were then blotted and negatively stained with 1% uranyl acetate for 1 min. Grids were examined using an 80 kV JEM-1400 electron microscope (JEOL Inc., Peabody, Massachusetts, United States) and images acquired with a digital camera (Gatan Orius, Gatan Inc., Pleasanton, California, United States).

##### 2.3.2.2 Cryo-Electron Microscopy

To analyze the morphology of EVs by cryo-EM, 3 µL of EV sample was first deposited onto a glow-discharged 200-mesh lacey carbon grid. Prior to freezing, the grid was loaded into the thermostatic chamber of a Leica EM-GP automatic plunge Freezer, set to 20°C and 95% humidity. Excess solution was blotted from the grid for 1–2 s with a Whatman n°1 filter paper and the grid immediately flash frozen in liquid ethane cooled to −185°C. Specimens were then transferred into a Gatan 626 cryo-holder. Cryo EM was carried out using a Jeol 2,100 microscope equipped with a LaB6 cathode operating at 200 kV under low-dose conditions. Images were acquired using SerialEM software (Mastronarde, 2005), with the defocus ranging from 600 to 1,000 nm, using a Gatan US4000 CCD camera. This device was placed at the end of a GIF quantum energy filter (Gatan Inc. Berwyn, Pennsylvania, United States) operating in zero-energy-loss mode, with a slit width of 25 eV. Images were recorded at a magnification corresponding to a calibrated pixel size of 0.87 Å.

##### 2.3.2.3 Nanoparticle Tracking Analysis (NTA)

EVs were resuspended in 50 µL D-PBS. Particle-size distribution and concentration were analyzed using a NanoSight LM10-HS instrument (Malvern Instruments Ltd., Malvern, United Kingdom) according to the manufacturer’s instructions. Briefly, EV samples were diluted 100-fold in D-PBS and the diluted preparation injected into the chamber. Samples were analyzed at room temperature for 60 s. Three replicates were performed for each sample. Data were acquired and analyzed using NTA 2.2 Build 127 software (Malvern Instruments Ltd., Malvern, United Kingdom).

##### 2.3.2.4 Specific Extracellular Vesicle Markers

EVs were characterized by analyzing tetraspanin (CD81, CD9) and β-actin protein expression by western blotting as recommended by the International Society for Extracellular Vesicles (ISEV) (Théry et al., 2018).

##### 2.3.2.5 Measurement of Acetylcholinesterase Activity

Acetylcholinesterase activity was measured using a colorimetric assay as previously described by Ellman et al. (1961). Briefly, 200 µL D-PBS containing 1 mM acetylcholine and 0.1 mM 5, 5′-Dithiobis 2-nitrobenzoic acid (DNTB) was added to 100 µL EV sample. After a 15 min incubation at room temperature, the absorbance was read at 450 nm using an Envision microplate reader. Data are expressed as the percentage difference in absorbance compared to the control (assay diluent D-PBS).

### 2.4 Biochemical Assays

#### 2.4.1 Cell Viability Assay

Cell viability was assessed using the WST-1 assay. Cells were seeded in 96-well plates at a density of 7,500 cells/well. After 48 h, cells were treated with LPS-EK or macrophage-derived EVs for 6 or 24 h, respectively. Ten percent dimethyl sulfoxide (DMSO) was used as a positive control for viability loss. The medium was then changed and the cells incubated in 100 µL DMEM 6546 medium containing 10 µL WST-1 reagent for 1 h at 37°C. Absorbance was measured at 450 nm using an Envision microplate reader (Perkin Elmer^®^, Waltham, Massachusetts, United States).

#### 2.4.2 Calcification Assay

VSMC calcification was assessed by measuring the intracellular calcium concentration using the o-cresolphthalein assay as previously described (Ray Sarkar and Chauhan, 1967). The total cell protein concentration was assessed by the method of Peterson (Peterson, 1977) and used to normalize the intracellular calcium concentration.

#### 2.4.3 Measurement of Oxidative Stress (ROS, O2•^−^, and NO Production)

The three fluorescent probes DCFH-DA, DHE, and DAF were used to measure ROS, O2•^−^, and NO production, respectively. Macrophages were seeded in white 96-well plates at a density of 7,500 cells/well. After 48 h, cells were washed twice with D-PBS and incubated at 37°C with 10 µM DCFH-DA for 30 min and then 10 µM DHE or 0.1 µM DAF for 1 h. Next, cells were washed twice with D-PBS and treated with LPS-EK or macrophage-derived EVs. Hydrogen peroxide (H_2_O_2_) at 50 and 500 µM was used as a positive control of ROS production. For certain experiments, the antioxidants N-acetyl-L-cysteine (NAC, 10 mM) and α-tocopherol (vitamin E, 10 µg/ml) were added to each well 1 h prior to treatment. For NO production, the iNOS substrate L-arginine was added to each well at 50 µM. All solutions were prepared in Krebs-Ringer-phosphate buffer (KRP). Fluorescence was measured using an Envision microplate reader (λ_Ex_ 492 nm, λ_Em_ 535 nm for DCFH-DA and DAF; λ_Ex_ 492 nm, λ_Em_ 615 nm for DHE).

#### 2.4.4 Western Blot Analysis

After treatment, cells were washed twice with cold D-PBS and lysed with RIPA buffer, sonicated, and centrifuged (16,000 × g, 5 min, 4°C). Supernatants were collected in a new tube and the protein concentration determined using the BCA^™^ Protein Assay kit according to the manufacturer’s instructions. Proteins were precipitated with methanol/chloroform (1/0.25; v/v) and centrifuged for 5 min at 16,000 × g at room temperature. The pellet was then resuspended in 4X Laemmli buffer and heated to 99°C for 5 min. Fifty µg of each protein sample was separated on a 12% SDS-PAGE gel and transferred onto a nitrocellulose membrane. After blocking, membranes were incubated overnight at 4°C with primary antibodies: rabbit anti-CD9 polyclonal antibody (1/1,000, GeneTex^®^ GTX55564), goat anti-CD81 polyclonal antibody (1/1,000, Santa Cruz Biotechnology, Inc.^®^ sc-31234), mouse monoclonal anti-β-actin antibody (1/5,000, Sigma-Aldrich^®^ A1978, clone AC-15), rabbit anti-SMPD3 (1/10,000, Sigma-Aldrich^®^ SAB2102245), or rabbit anti-p62/SQSTM1 (1/1,000, Sigma-Aldrich^®^ P0067). After several washes, membranes were incubated with goat anti-rabbit IgG-HRP (1/5,000, Santa Cruz Biotechnology, Inc.^®^ sc-2004), goat anti-mouse IgG-HRP (1/5,000, Santa Cruz Biotechnology, Inc.^®^, sc-2005), or rabbit anti-goat IgG (H + L)-HRP (1/5,000, Southern Biotech^®^, 6,160-05) for 1 h at room temperature. Then, proteins were visualized using ECL^™^ Western Blot Detection Reagents and a ChemiDoc^™^ MP Imaging System (Bio-Rad^®^, Hercules, California, United States). β-actin protein levels were quantified to normalize protein levels.

### 2.5 RNA Extraction and Quantitative Real-Time PCR

After treatment, cells were washed twice with D-PBS. RNA extraction was then performed using a mixture of TRIzol^™^ and chloroform (1/0.2; v/v). After a 15-min centrifugation (12,000 × g, 4°C), RNA was collected and precipitated with isopropanol. The RNA pellet was then washed twice with 70% ethanol and resuspended in 40 µL RNase/DNase-free water. The RNA concentration was determined using a NanoVue™ Plus device (Thermo Fisher Scientific^®^, Waltham, Massachusetts, United States). cDNA was synthesized using a High-Capacity RNA-to-cDNA^™^ kit according to the manufacturer’s instructions. Quantitative real-time PCR was carried out using Takyon^™^ and specific primers (Supplementary Table S1). A CFX96 Touch Real-Time PCR Detection System (Bio-Rad^®^, Hercules, California, United States) was used with the following steps: 95°C for 15 s, followed by 40 cycles of 95°C for 15 s, 60°C for 1 min and 72°C for 30 s. β-actin was used as a housekeeping gene to normalize gene expression.

### 2.6 MSD Multiplex Immunoassay

Proinflammatory cytokine protein levels were measured in macrophages and EVs derived from macrophages using a proinflammatory panel multiplex kit (V-PLEX^®^ K15048D, Meso Scale Diagnostics^®^, Rockville, Maryland, United States) according to the manufacturer’s instructions. Briefly, 50 µL/well of sample or calibrators was added to a 96-well plate pre-coated with capture antibodies. After a 2-h incubation with shaking at room temperature and three washes (D-PBS with 0.05% Tween 20), 25 µL 1X detection antibody solution was added to each well. After another 2-h incubation under the same conditions and three washes, 150 µL 2X Read Buffer T was added to each well. The plate was then analyzed using the MSD instrument (Meso Scale Diagnostics^®^, Rockville, Maryland, United States).

### 2.7 Mass Spectrometry Proteomic Analysis

#### 2.7.1 Proteomic Digestion for Mass Spectrometry

An S-TrapTM microspin column digestion was performed on 10 µg of macrophage-derived EVs according to the manufacturer’s instructions (Protifi, Hutington, United States). Briefly, samples were reduced with 20 mM tris (2-carboxyethyl) phosphine and then alkylated with 50 mM chloroacetamide for 15 min at room temperature. Aqueous phosphoric acid was then added to a final concentration of 1.2%, followed by the addition of S-Trap binding buffer (90% aqueous methanol, 100 mM tetraethylammonium bromide, pH 7.1). The mixtures were then loaded onto S-Trap columns. Two extra washing steps were performed to eliminate SDS. Samples were then digested with 1 µg trypsin (Promega, Madison, Wisconsin, United States) at 47°C for 1 h. After elution, peptides were vacuum dried and resuspended in 45 µL 2% acetonitrile/0.1% formic acid mixture in HPLC-grade water prior to MS analysis. A volume of 1 µL of the peptide suspension was injected into a nanoelute high-performance liquid chromatography (HPLC) system coupled to a timsTOF Pro mass spectrometer (Bruker Daltonics, Germany). HPLC separation was performed using a mixture of 0.1% formic acid in water, 2% acetonitrile (Solvent A), and 0.1% formic acid in acetonitrile (Solvent B) at 250 nL/min using a packed emitter column (C18, 25 cm × 75 μm 1.6 μm) (Ion Optics, Australia) with a gradient elution (2–11% solvent B ove r19 min, 11–16% over 7 min, 16–25% over 4 min, 25–80% over 3 min, and, finally, 80% for 7 min to wash the column). Mass-spectrometric data were acquired using the parallel accumulation serial fragmentation (PASEF) acquisition method. The measurements were carried out over an m/z range from 100 to 1700 Th, with ion mobility from 0.8 to 1.3 V s/cm^2^ (1/k0). The total cycle time was set to 1.2 s and the number of PASEF MS/MS scans was set to 10. A total of 2,762 proteins was identified in at least 60% of all samples.

The mass spectrometry proteomics data have been deposited in the ProteomeXchange Consortium via the PRIDE (Perez-Riverol et al., 2019) partner repository with the dataset identifier PXD029441 and 10.6019/PXD029441.

#### 2.7.2 Data Analysis

The data were analyzed using MaxQuant version 1.6.14.0 (Max-Planck, Munich, Germany) and searched using the Andromeda search engine against the UniProtKB/Swiss-Prot *Mus musculus* database (release 02-04-2020, 17040 entries). Mass deviations of 3 and 20 ppm were used to search parent mass and fragment ions, respectively. The minimum peptide length was set to seven amino acids and strict specificity for trypsin cleavage was required, allowing up to two missed cleavage sites. Carbamidomethylation (Cys) was set as a fixed modification, whereas oxidation (Met) and N-term acetylation were set as variable modifications. The false discovery rate (FDR) for both proteins and peptides was set to 1%. Scores were calculated in MaxQuant as previously described (Cox and Mann, 2008). The reverse and common contaminant hits were removed from the MaxQuant output. Proteins were quantified according to the MaxQuant label-free algorithm using LFQ intensities; protein quantification was obtained using at least two peptides per protein. Matches between runs were allowed. Statistical and bioinformatic analysis, including heatmaps, profile plots, and clustering, were performed using Perseus software (version 1.6.14.0) freely available at www.perseus-framework.org (Tyanova et al., 2016). For statistical comparisons, we set up five groups, each containing up to five biological replicates. We then filtered the data to keep only proteins with at least four valid values in at least one group. Next, the data were imputed to fill missing data points by creating a Gaussian distribution of random numbers with a standard deviation of 33% relative to the standard deviation of the measured values and a 1.8 standard deviation downshift of the mean to simulate the distribution of low-signal values. We performed an ANOVA test, FDR <0.05, S0 = 0.5. Hierarchical clustering of proteins that survived the test was performed using Perseus on log-transformed LFQ intensities after z-score normalization of the data using Euclidean distances.

### 2.8 Statistical Analysis

Statistical analyses were performed using GraphPad Prism (version 7.0, San Diego, California, United States). Wilcoxon-Mann-Whitney and Kruskal–Wallis tests were performed with a significance threshold of 0.05. Data are expressed as the mean ± standard error of mean (SEM) from at least three independent experiments.

Principal Component Analysis has been performed on log transformed and scaled values from targeted MSD multiplex immunoassay using the R package pcaMethods.

## 3 Results

### 3.1 Effects of LPS-EK on Macrophages

#### 3.1.1 Lipopolysaccharide From *Escherichia coli K12* Induces Inflammation in Macrophages

**FIGURE 1:**
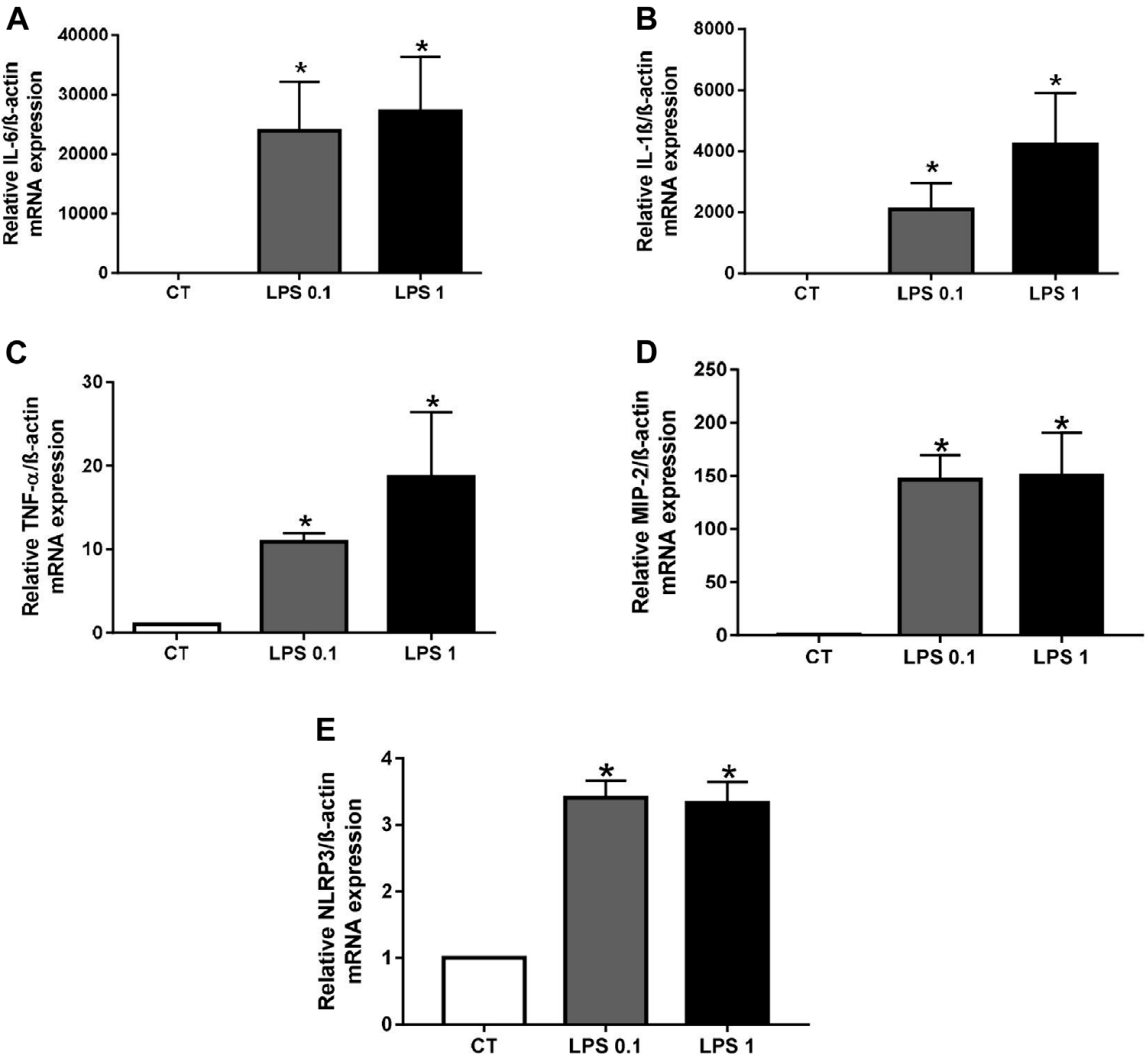
Lipopolysaccharide-EK (LPS-EK) induces inflammation in macrophages. RAW cells were incubated with LPS-EK for 6 h. Gene expression of **(A)** IL-6, **(B)** IL-1β, **(C)** TNF-α, **(D)** MIP-2, and **(E)** NLRP3 was quantified by quantitative real-time PCR and normalized to that of the housekeeping gene ß-actin. Data are expressed as the mean ± SEM of four independent experiments performed in triplicate (*n* = 4). *p < 0.05 *vs*. CT, Mann-Whitney test. LPS 0.1: 0.1 µg/ml LPS-EK, LPS 1: 1 µg/ml LPS-EK.

In setting up our cellular model of inflammation, we first verified the effect of LPS-EK on inflammation in RAW macrophages by measuring the mRNA levels of various pro-inflammatory cytokines (IL-6, IL-1β, TNF-α, and MIP-2), as well as that of the NLRP3 inflammasome marker, before and after treatment. The mRNA levels of these markers were all significantly higher after LPS-EK treatment than those of unstimulated macrophages (CT) (*p < 0.05 *vs.* CT, Figure 1). We also analyzed the levels of 10 proinflammatory cytokines in LPS-EK-treated or untreated macrophages using an MSD multiplex kit. Macrophages treated with LPS-EK showed significantly higher levels of these proinflammatory cytokines than untreated control cells (*p < 0.05 *vs.* CT, Supplementary Figure S2). These results show that LPS-EK induces an inflammatory response in our cellular model.

#### 3.1.2 Lipopolysaccharide From *Escherichia coli K12* Induces Oxidative Stress in Macrophages

**FIGURE 2:**
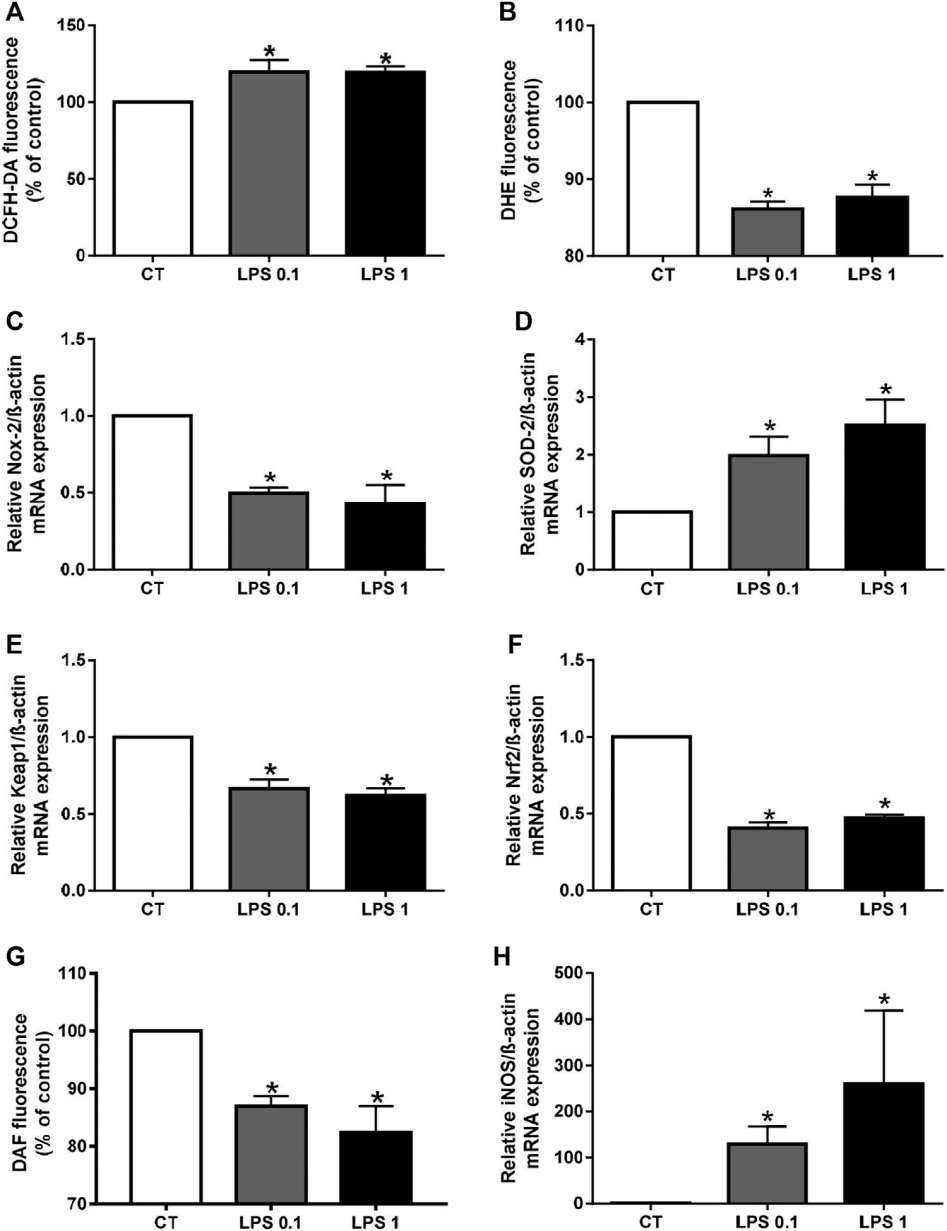
Lipopolysaccharide-EK (LPS-EK) induces oxidative stress in macrophages. ROS **(A)**, O2^•–^ **(B)**, and NO **(G)** production in RAW cells was measured using the fluorescent probes DCF, DHE, and DAF, respectively. Gene expression of **(C)** Nox-2, **(D)** SOD-2, **(E)** Keap1, **(F)** Nrf2, and **(H)** iNOS was quantified by quantitative real-time PCR and normalized to that of the housekeeping gene ß-actin. Data are expressed as the mean ± SEM of four independent experiments performed in triplicate (*n* = 4). *p < 0.05 *vs*. CT, Mann-Whitney test. LPS 0.1: 0.1 µg/ml LPS-EK, LPS 1: 1 µg/ml LPS-EK.

As oxidative stress is also involved in the calcification process, we next determined the effect of LPS on reactive oxygen species (ROS) production using the fluorescent probe DCFH-DA. ROS production by cells treated with LPS-EK was significantly higher than that of untreated cells (*p < 0.05 *vs*. CT) (Figure 2A). In addition, mRNA levels of two components of the antioxidant system, Keap1 and Nrf2, were lower after LPS-EK treatment, which could partially explain the increase in oxidative stress (*p < 0.05 *vs*. CT, Figures 2E,F). Among the various ROS, superoxide anions (O_2_^•–^) play an important role in oxidative stress. We therefore investigated the effect of LPS-EK on O_2_^•–^ production using the fluorescent probe DHE. Surprisingly, O_2_^•–^ production was significantly lower in LPS-EK treated cells than in untreated cells (*p < 0.05 *vs*. CT, Figure 2B). We investigated this seeming discrepancy by measuring the mRNA levels for both Nox-2, the enzyme that synthesizes O_2_^•–^, and superoxide dismutase (SOD-2), which is responsible for O_2_^•–^ degradation. We observed significantly higher Nox-2 and lower SOD-2 mRNA levels in LPS-EK stimulated macrophages than in untreated macrophages (*p < 0.05 *vs*. CT, Figures 2C,D). Our results thus show that LPS-EK decreases superoxide anion production by decreasing Nox-2 expression and increasing SOD-2 expression. Superoxide anions can rapidly react with nitric oxide (NO) to form peroxynitrite (ONOO^−^). Thus, we next investigated the effect of LPS-EK on NO production using the fluorescent probe DAF. NO production was significantly lower after LPS-EK treatment than in untreated cells (*p* < 0.05 *vs*. CT, Figure 2G), despite significantly higher levels of iNOS (*p < 0.05 *vs*. CT, Figure 2H). These results suggest that both decreases in NO and O_2_^•–^ production may be partially explained by the formation of peroxynitrite.

#### 3.1.3 Lipopolysaccharide From *Escherichia coli K12* Decreases Extracellular Vesicle Biogenesis in Macrophages

**FIGURE 3:**
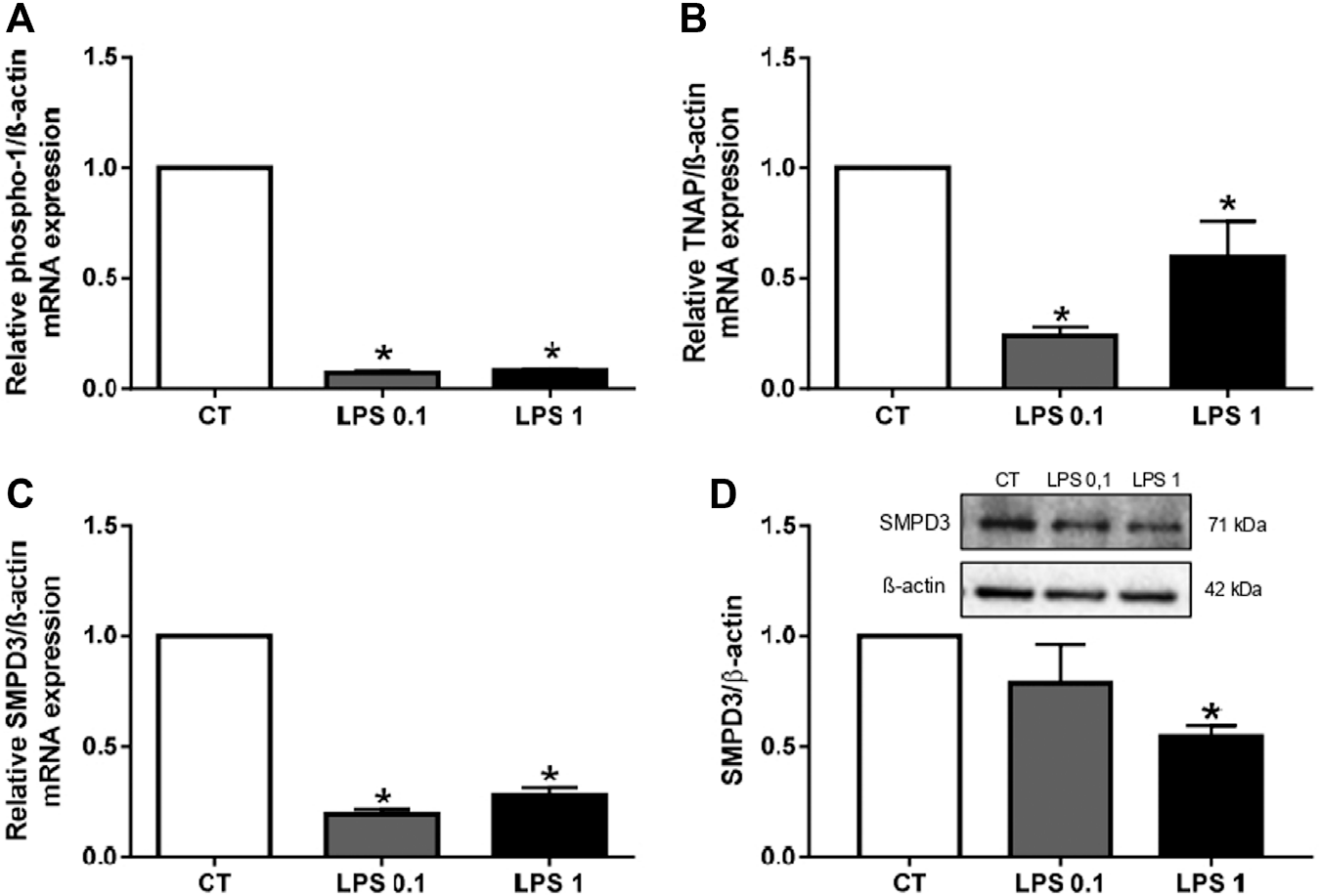
Lipopolysaccharide-EK (LPS-EK) decreases EV biogenesis in macrophages. RAW cells were incubated with LPS-EK for 6 h. Gene expression of EV of the biogenesis markers **(A)** phospho-1, **(B)** TNAP, and **(C)** SMPD3 was then quantified by RT-qPCR and normalized to that of the housekeeping gene ß-actin. **(D)** SMPD3 protein expression was studied by western blotting and normalized to that of the housekeeping gene ß-actin. Data are expressed as the mean ± SEM of four independent experiments performed in triplicate (*n* = 4). *p < 0.05 *vs*. CT, Mann-Whitney test. LPS 0.1: 0.1 µg/ml LPS-EK, LPS 1: 1 µg/ml LPS-EK.

The aim of this study was to determine whether EVs can propagate inflammation and oxidative stress to recipient cells. Thus, we first studied EV biogenesis in RAW cells after LPS-EK treatment by analyzing the mRNA levels of various EV biogenesis markers (phospho-1, TNAP, and SMPD3) in LPS-EK-treated macrophages. The mRNA levels of these markers were all significantly lower in LPS-EK-treated cells than in unstimulated macrophages (*p < 0.05 *vs*. CT, Figures 3A–C), suggesting a decrease in EV biogenesis. SMPD3 protein levels were also significantly lower after 1 µg/ml LPS-EK treatment than in untreated macrophages (*p < 0.05 *vs*. CT, Figure 3D).

#### 3.1.4 Lipopolysaccharide From *Escherichia coli K12* Decreases Autophagy in Macrophages

**FIGURE 4:**
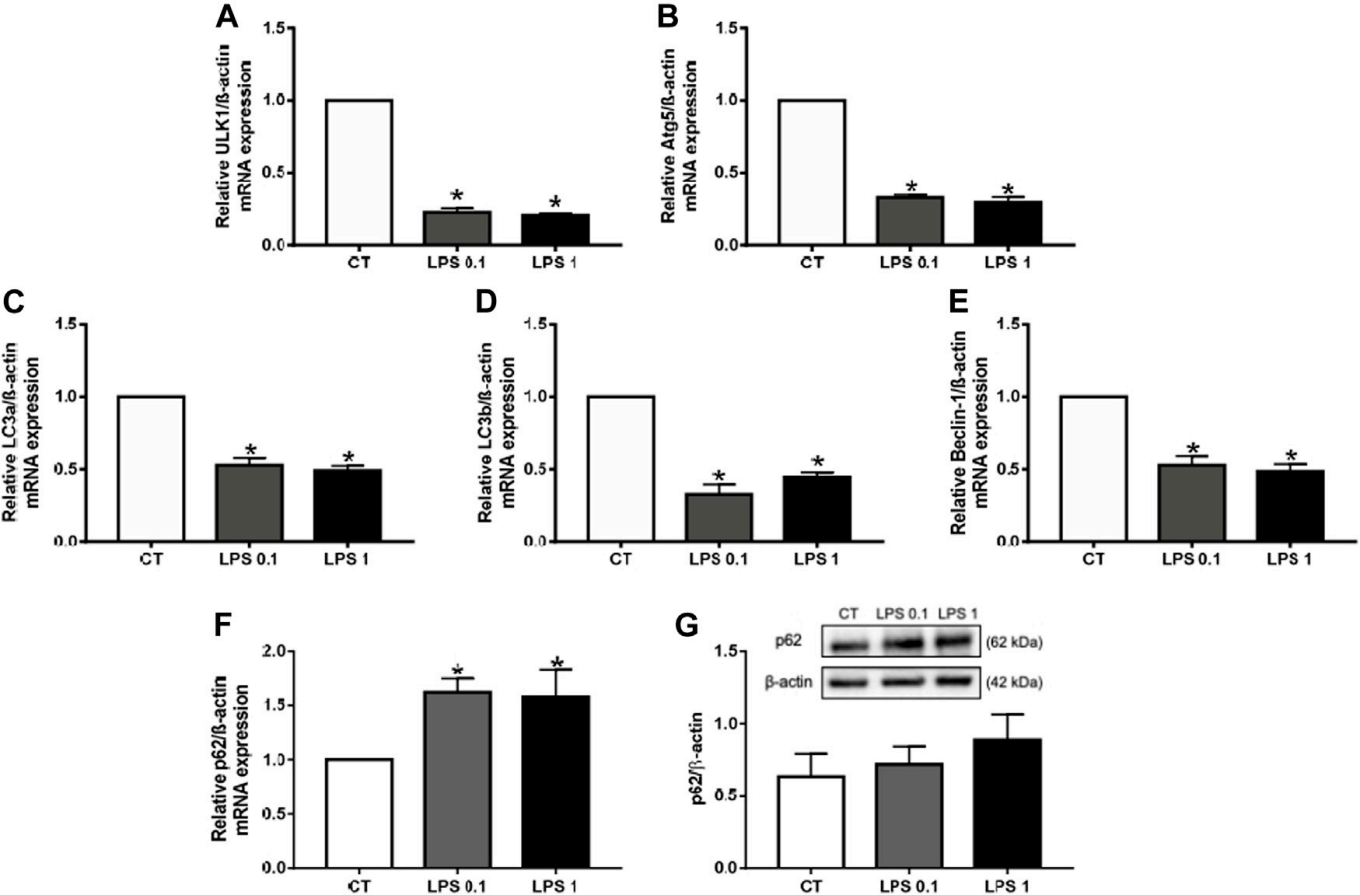
Lipopolysaccharide-EK (LPS-EK) decreases autophagy in macrophages. RAW cells were incubated with LPS-EK for 6 h. Gene expression of the autophagy markers **(A)** ULK1, **(B)** Atg5, **(C)** LC3a, **(D)** LC3b, **(E)** Beclin-1, and **(F)** p62 was then quantified by RT-qPCR and normalized to that of the housekeeping gene ß-actin. **(G)** p62 protein expression was studied by western blotting and normalized to that of the housekeeping gene ß-actin. Data are expressed as the mean ± SEM of four independent experiments performed in triplicate (*n* = 4). *p < 0.05 *vs*. CT, Mann-Whitney test. LPS 0.1: 0.1 µg/ml LPS-EK, LPS 1: 1 µg/ml LPS-EK.

Recent studies have highlighted novel functions of autophagy in the biogenesis and secretion of EVs (Yang et al., 2021). Indeed, it has been shown that not only exosome biogenesis and autophagy share molecular machinery but also that substantial crosstalk exists between these two processes. We therefore analyzed the effect of LPS-EK on autophagy markers, such as ULK1, Beclin-1, Atg5, LC3a, and LC3b. The mRNA levels of all these markers were significantly lower after LPS-EK treatment than in unstimulated macrophages (*p < 0.05 *vs*. CT, Figures 4A–E), suggesting a decrease in the autophagy process. p62, also known as sequestome-1 (SQSTM), is an autophagy cargo receptor (Lamark et al., 2009) that can be used as a sensor of autophagic flux. Indeed, p62 accumulates when autophagy is inhibited and decreased levels of p62 can be observed when autophagy is induced. After LPS-EK treatment, p62 mRNA levels were higher than in unstimulated macrophages (*p < 0.05 *vs*. CT, Figure 4F), confirming inhibition of the autophagic flux. p62 protein levels were not modified after LPS-EK treatment relative to untreated cells (Figure 4G). Overall, our results show that LPS-EK not only induces inflammation and oxidative stress in RAW cells but also decreases autophagy and EV biogenesis.

### 3.2 Effects of EV-LPS on Smooth Muscle Cells

#### 3.2.1 Characterization of Macrophage-Derived EVs

**FIGURE 5:**
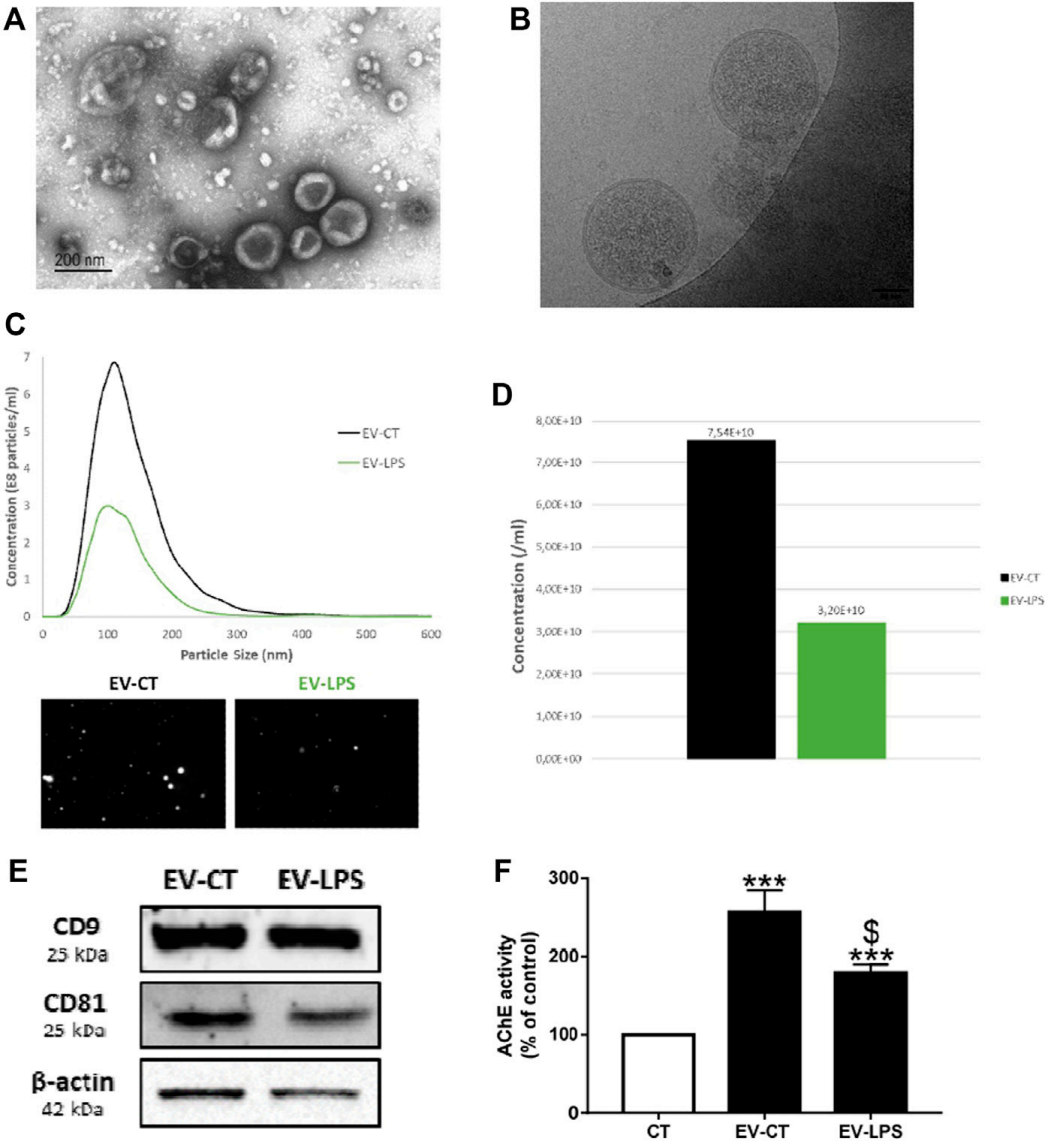
Characterization of macrophage-derived EVs. EVs were isolated from the culture media of RAW cells incubated with (EV-LPS) or without (EV-CT) 1 µg/ml lipopolysaccharide-EK (LPS-EK) for 6 h. Evaluation of EV morphology by **(A)** transmission electron microscopy (TEM) and **(B)** cryo-electron microscopy (cryo-EM). **(C)** Particle-size distribution and **(D)** the total concentration of macrophage-derived EVs were measured by nanoparticle-tracking analysis (NTA). **(E)** CD9, CD81, and β-actin protein expression were assessed by western blotting. **(F)** Quantification of the enzymatic activity of acetylcholinesterase (AChE) in macrophage-derived EVs. Control AChE activity in the assay diluent 1X-D-PBS (CT) was defined as 100%. Data are expressed as the mean ± SEM of seven independent experiments performed in triplicate (*n* = 7). ***p < 0.001 *vs*. CT; $*p* < 0.05 *vs.* EV-CT, Mann-Whitney test.

We characterized macrophage-derived EVs by their morphology, size, and concentration (Figure 5) using transmission electron microscopy (TEM) (Figure 5A), cryo-electron microscopy (cryo-EM) (Figure 5B), and nanoparticle-tracking analyses (NTA) (Figure 5C). The particle size for exosomes is between 50 and 150 nm and between 50 and 500 nm for microvesicles (van Niel et al., 2018). The average particle size detected by NTA was 139 nm for EVs isolated from untreated macrophages (EV-CT) and 130 nm for EVs isolated from LPS-EK-treated macrophages (EV-LPS) (Figure 5C). Thus, our EV samples were likely enriched for both exosomes and microvesicles. Furthermore, we verified EV membrane integrity by cryo-EM (Figure 5B). Indeed, we could observe a discernible lipid bilayer and internal vesicular structures (Figure 5B). The diameter of EVs can vary, as well as the content. The International Society for Extracellular Vesicles (ISEV) has published recommendations for EV characterization (Théry et al., 2018). Indeed, two categories of markers must be analyzed in sample preparations to confirm the presence of EVs: transmembrane proteins, such as tetraspanins, and cytosolic proteins recovered in EVs (Théry et al., 2018). Thus, we performed western blotting to determine the presence of EV-specific tetraspanin (CD9, CD81) and β-actin and detected all of these EV markers in our EV samples (Figure 5E). Acetylcholinesterase (AChE) is expressed in macrophages (Fujii et al., 2017) and can also be found in macrophage-derived EVs. According to the ISEV, AChE activity can also be used to characterize EVs (Figure 5F). Overall, these results confirm the presence of EVs in both preparations. LPS-EK treatment of macrophages resulted in a lower number of EVs than in control untreated cells (Figure 5D). Indeed, we detected 7.54 × 10^10^ particles in the EV-CT sample, whereas the EV-LPS preparation contained 3.20 × 10^10^ particles. Furthermore, we observed significantly less AChE in the EV-LPS preparation than in the EV-CT preparation. Both results could be explained by the lower amount of secreted EVs due to decreased EV biogenesis after LPS-EK treatment ($*p* < 0.05 *vs*. EV-CT, Figure 5F). Importantly, the EVs were isolated from the same volume of cell-culture media.

#### 3.2.2 Effect of Macrophage-Derived EVs on MOVAS Cell Viability

**FIGURE 6:**
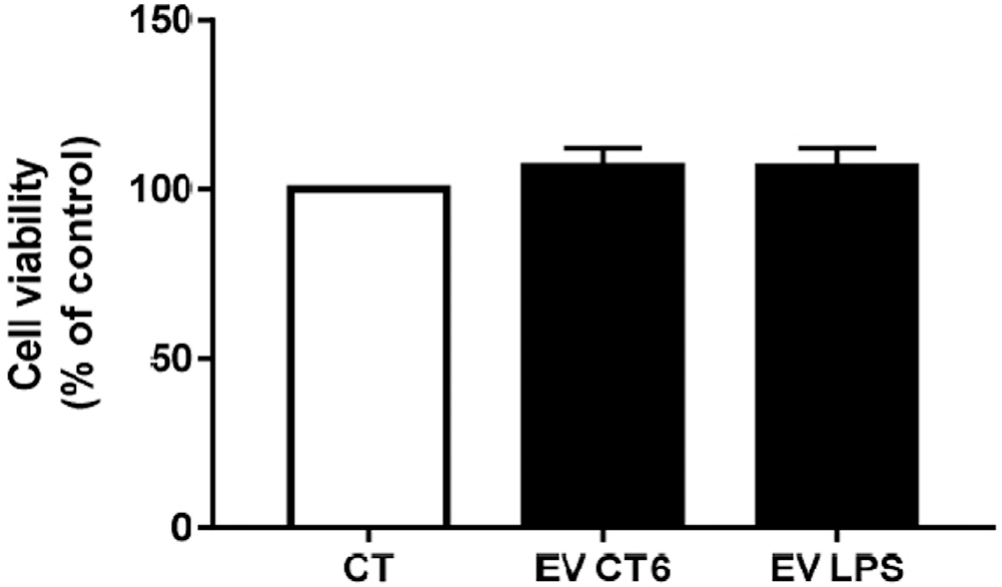
Effect of macrophage-derived EVs on smooth muscle cell viability. MOVAS-1 cells were incubated for 24 h with EVs secreted by either treated (1 µg/ml LPS-EK; EV-LPS) or untreated macrophages (EV-CT) or without (CT). Cell viability was measured using the WST-1 assay. The viability of cells incubated without EVs (CT) was defined as 100%. Data are expressed as the mean ± SEM of four independent experiments performed in triplicate (*n* = 4). *p* < 0.05 is considered significant, Wilcoxon- Mann-Whitney test.

We next studied the effects of macrophage-derived EVs on the smooth muscle cell line MOVAS-1. Cell viability was first assessed after 24 h of treatment with macrophage-derived EVs. EVs secreted by macrophages did not affect smooth muscle cell viability relative to untreated cells (CT), regardless of the treatment (Figure 6).

#### 3.2.3 EV-LPS Induce Inflammation in Smooth Muscle Cells

**FIGURE 7:**
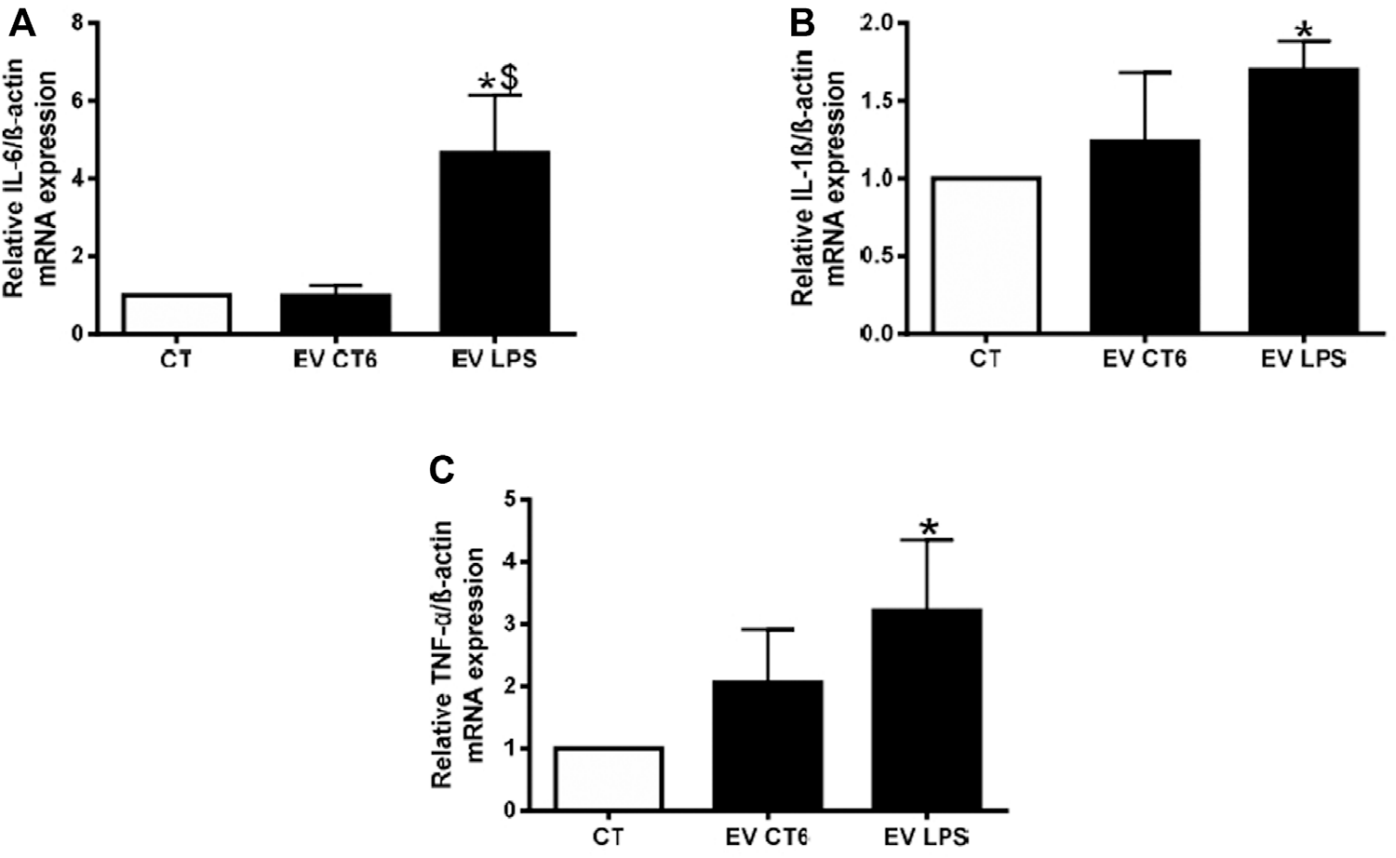
EV-LPS increases cytokine gene expression in smooth muscle cells. MOVAS-1 cells were incubated with RAW cell-derived EVs for 24 h. Gene expression of **(A)** IL-6, **(B)** IL-1ß, and **(C)** TNF-α was then quantified by quantitative real-time PCR and normalized to that of the housekeeping gene ß-actin. Data are expressed as the mean ± SEM of four independent experiments performed in triplicate (*n* = 4). *p < 0.05 *vs*. CT, $*p* < 0.05 *vs*. EV-CT, Mann-Whitney test. EV-CT: EV-derived from untreated macrophages, EV-LPS: EV-derived from LPS-EK-treated macrophages.

Our objective was to study the propagation of inflammation from macrophages to smooth muscle cells. Thus, we measured the mRNA levels of proinflammatory cytokines (IL-6, IL-1ß, and TNF-α) in MOVAS cells treated with macrophage-derived EVs. We observed significantly higher proinflammatory cytokine mRNA levels after 24 h of treatment of smooth muscle cells with EV-LPS than in untreated cells (CT) (*p < 0.05 *vs*. CT, Figure 7). There was no effect of EVs derived from control cells (EV-CT) on cytokine levels. A comparison of the effects of the two populations of EVs showed only the increase in IL-6 mRNA levels to be significant ($ *p* < 0.05 *vs*. EV-CT, Figure 7A). These results show that EV-LPS are able to induce inflammation mainly *via* an increase in IL-6 expression.

#### 3.2.4 EV-LPS Induces Oxidative Stress in Smooth Muscle Cells

**FIGURE 8:**
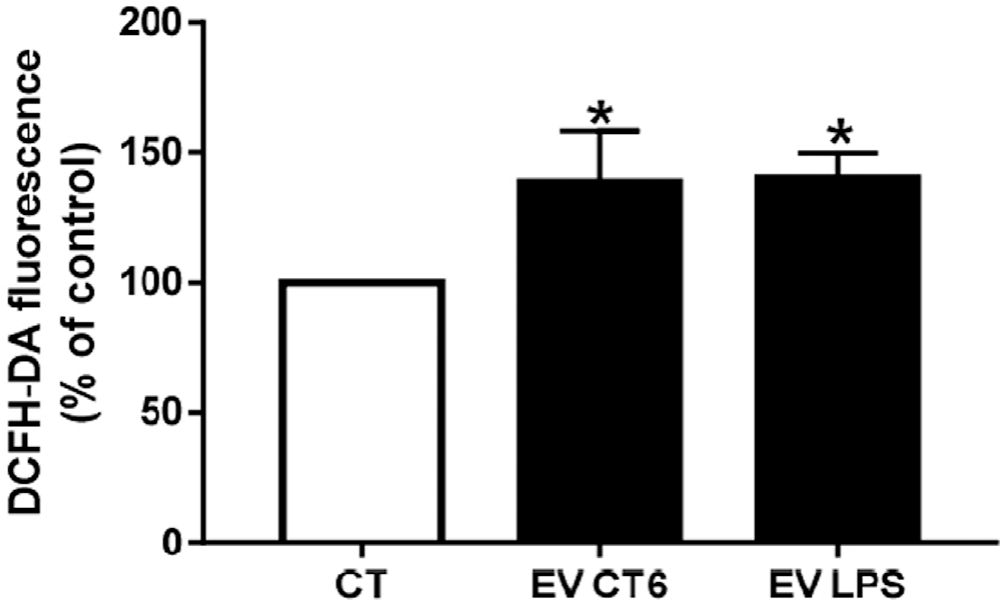
Macrophage-derived EVs induce ROS production in smooth muscle cells. MOVAS-1 cells were incubated with 10 µM DCFH-DA in D-PBS at 37°C for 30 min and then treated with macrophage-derived EVs for 24 h. ROS production was determined by measuring fluorescence (λEx 492 nm, λEm 535 nm). ROS production by untreated control cells (CT) was defined as 100%. Data are expressed as the mean ± SEM of four independent experiments performed in triplicate (*n* = 4). *p < 0.05 *vs*. CT, Mann-Whitney test. EV-CT: EV-derived from untreated macrophages, EV-LPS: EV-derived from LPS-EK-treated macrophages.

**FIGURE 9:**
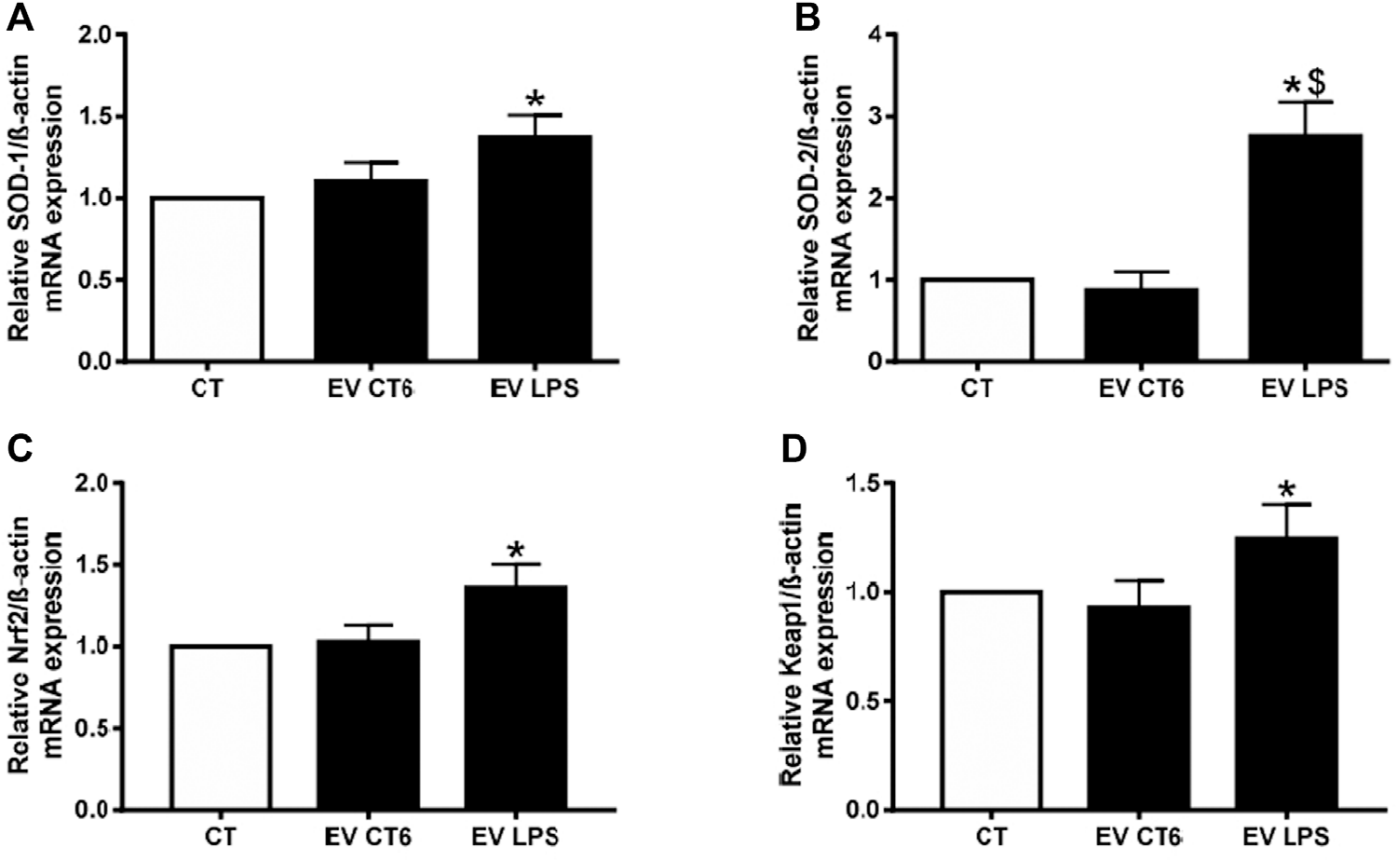
EV-LPS increase antioxidant marker gene expression in smooth muscle cells. MOVAS-1 cells were incubated with EVs-derived RAW cells for 24 h. Gene expression of **(A)** SOD-1, **(B)** SOD-2, **(C)** Nrf2, and **(D)** Keap1 was then quantified by quantitative real-time PCR and normalized to that of the housekeeping gene ß-actin. Data are expressed as the mean ± SEM of four independent experiments performed in triplicate (*n* = 4). *p < 0.05 *vs*. CT, $*p* < 0.05 *vs*. EV-CT, Mann-Whitney test. EV-CT: EV-derived from untreated macrophages, EV-LPS: EV-derived from LPS-EK-treated macrophages.

We investigated the effect of macrophage-derived EVs on oxidative stress in smooth muscle cells by measuring intracellular ROS production. Both EV preparations (EV-CT and EV-LPS) significantly increased ROS production over that of untreated smooth muscle cells (*p < 0.05 *vs*. CT, Figure 8). This result suggests that the effect of macrophage-derived EVs on oxidative stress in smooth muscle cells is independent of macrophage treatment. We next measured mRNA levels of antioxidant enzymes (SOD-1, SOD-2) and nuclear receptors (Nrf2, and Keap1) involved in the antioxidant response. Surprisingly, the mRNA levels of all markers were significantly higher in cells incubated with EV-LPS than in untreated cells (CT) (*p < 0.05 *vs*. CT, Figure 9). Only SOD-2 mRNA levels were higher in MOVAS cells treated with EV-LPS than those treated with EV-CT ($*p* < 0.05 *vs*. EV-CT, Figure 9B). Overall, these results suggest that EV-LPS can affect the oxidative stress response of smooth muscle cells.

#### 3.2.5 Analysis of the Content of EV-LPS

**FIGURE 10:**
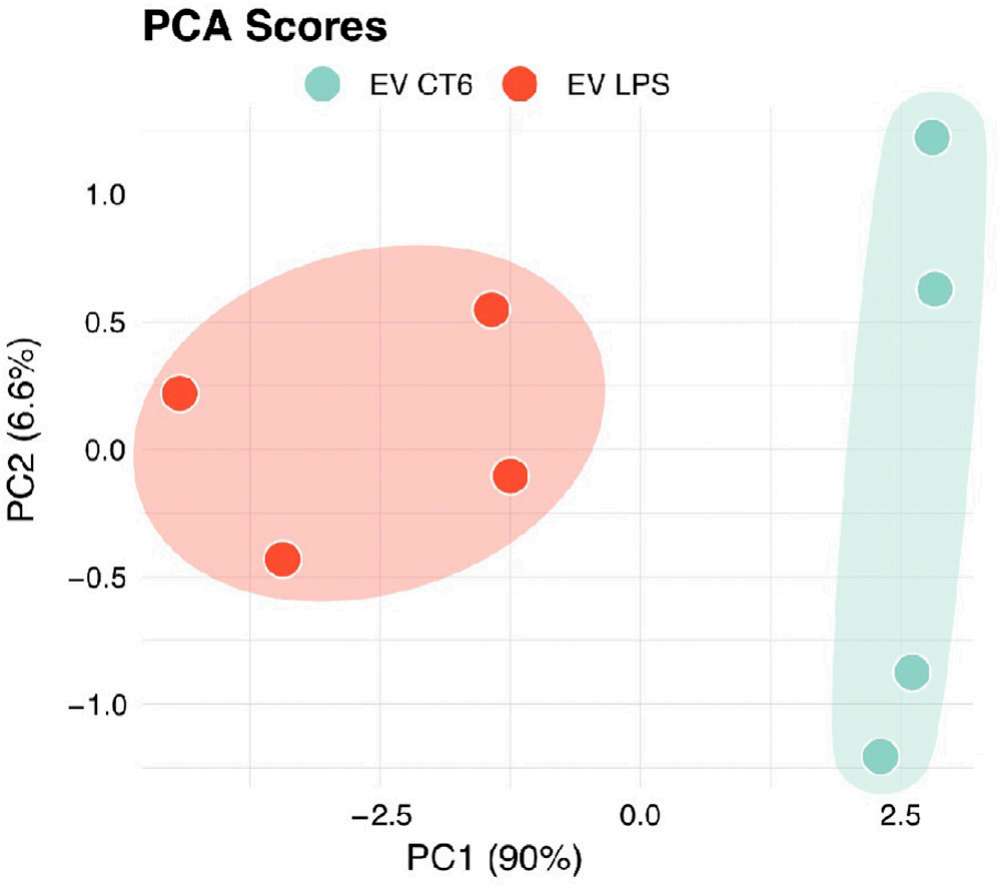
Principal component analysis score plot of EV-CT6 and EV-LPS. EVs were isolated from the culture media of RAW cells incubated with (EV-LPS) or without (EV-CT6) LPS-EK. Proinflammatory cytokine protein levels (IL-1ß, IL-2, IL-4, IL-5, IL-6, IL-10, IL-12p70, TNF-α, IFN-γ, and KC-GRO) were measured in EVs derived from the macrophages using an MSD multiplex immunoassay. PCA analysis score plot is performed using four independent experiments (*n* = 4).

**FIGURE 11:**
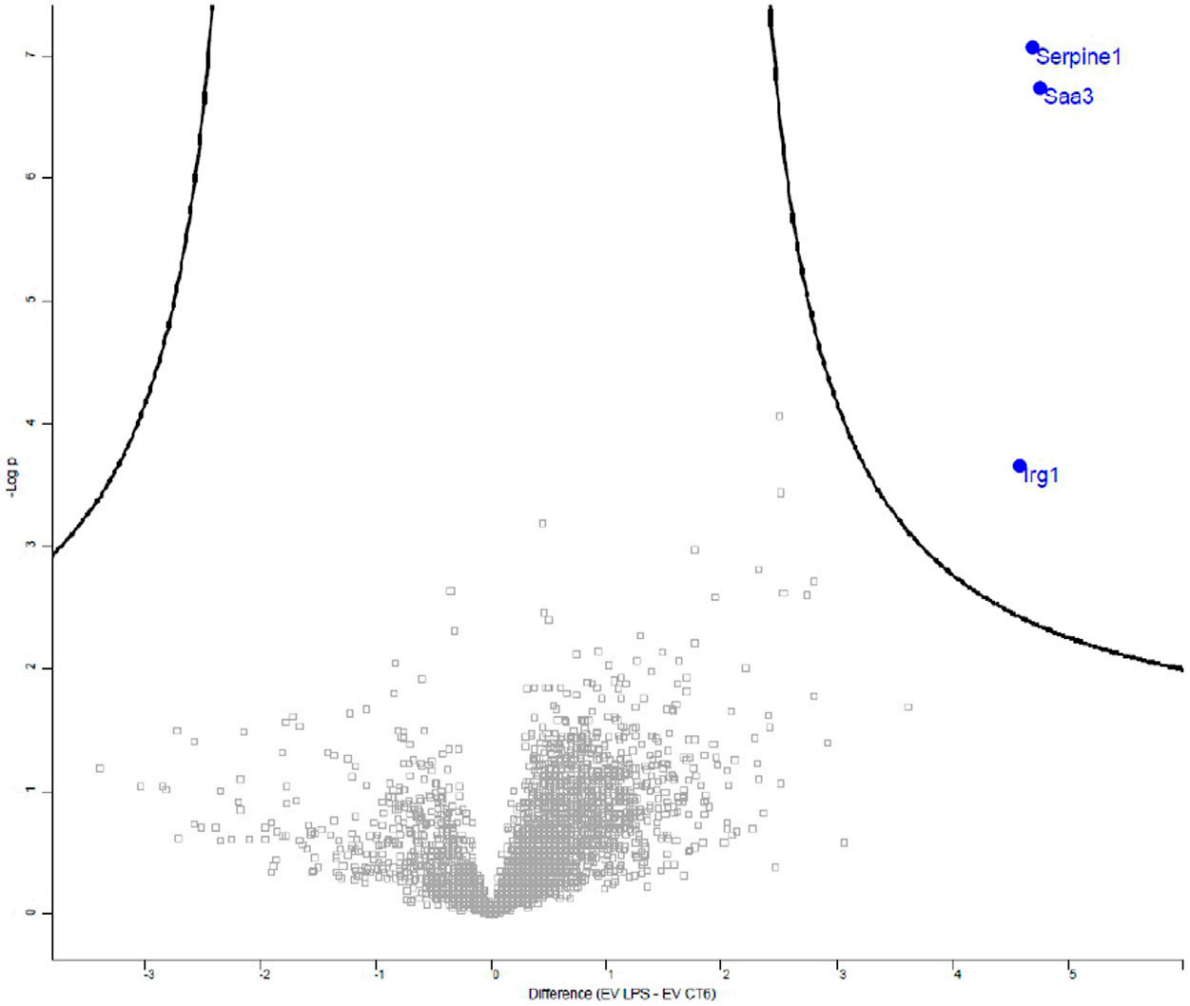
Volcano plot representing the effects of lipopolysaccharide-EK (LPS-EK) on the protein content of macrophage-derived extracellular vesicles (EVs). EVs were isolated from the culture media of RAW cells incubated with (EV-LPS) or without (EV-CT6) lipopolysaccharide-EK (LPS-EK). High-performance liquid chromatography (HPLC) coupled to mass spectrometry analysis was performed. An unpaired Student’s t test was conducted and a *p*-value < 0.01 and a false discovery rate (FDR) < 5% were used. Five independent experiments were performed.

Inflammation is known to play an important role in vascular calcification. We thus first analyzed the inflammatory content of EVs by measuring the expression of 10 proinflammatory cytokines in EV-LPS and EV-CT using an MSD multiplex kit. Proinflammatory cytokine levels were, indeed, significantly higher in EV-LPS than EV-CT6 (*p < 0.05 *vs.* CT, Supplementary Figure S3). We further analyzed the data using an unsupervised analysis approach to visualize any samples that clustered based on their inflammatory profile. The principal component analysis score plot showed a clear separation between the inflammatory profiles of EV-CT6 and EV-LPS (Figure 10). These results show that there is a specific inflammatory profile that distinguishes between these two conditions. We then analyzed the EV protein content by mass spectrometry and detected several EV-specific proteins in our samples, confirming the presence of EVs in our preparations (Supplementary Table S2). Furthermore, the level of three proteins in EV LPS were higher than in EV CT6: cis-aconitate decarboxylase (CAD), encoded by the *immunoresponsive gene 1* (*Irg1*); plasminogen activator inhibitor-1 (PAI-1), encoded by the *Serpine1* gene; and serum amyloid A-3 protein (Saa3), encoded by the *Saa3* gene (*p* < 0.01 *vs.* EV CT6, Figure 11).

#### 3.2.6 EV-LPS Increase Pi-Induced Calcification by Inducing the Osteogenic Switch of Smooth Muscle Cells

**FIGURE 12:**
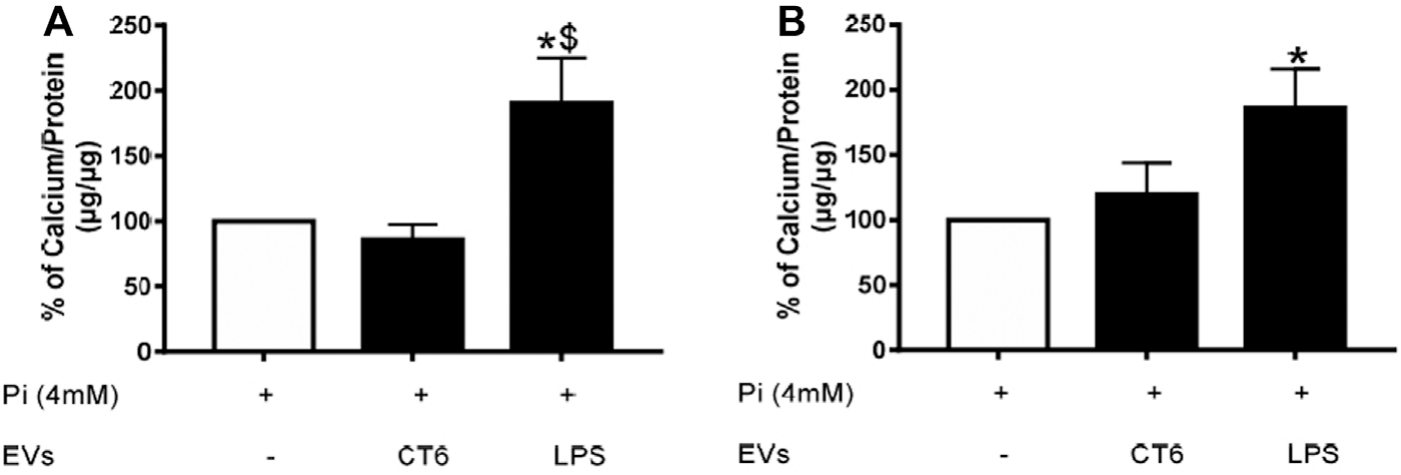
EV-LPS enhance Pi-induced calcification in smooth muscle cells. Calcification in MOVAS-1 cells was induced by incubation with 4 mM Pi with or without RAW cell-derived EVs for **(A)** 14 days or **(B)** during the last 7 days of a 14-day calcification induction. Calcification was then measured using the OCP method. Data are expressed as the mean ± SEM of four independent experiments performed in triplicate (*n* = 4). *p < 0.05 *vs.* 4 mM Pi, $*p* < 0.05 *vs.* EV-CT, Mann-Whitney test. EV-CT: EVs derived from untreated macrophages, EV-LPS: EVs derived from LPS-EK-treated macrophages.

**FIGURE 13:**
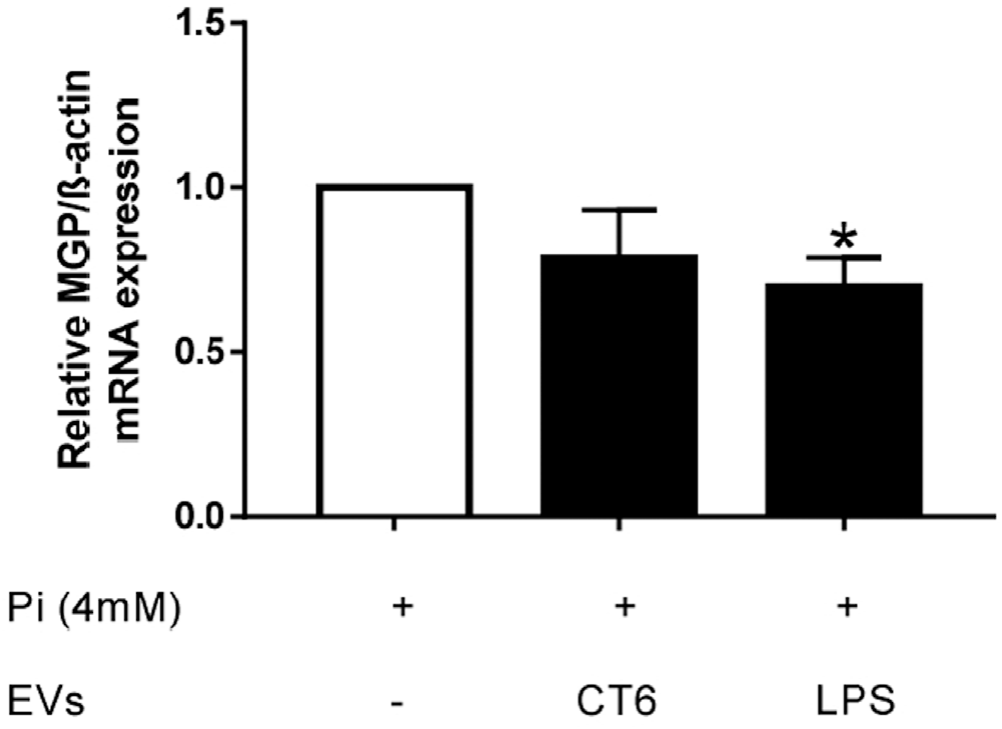
EV-LPS decrease MGP gene expression in smooth muscle cells. MOVAS-1 cells were incubated for 7 days with 4 mM Pi with or without EVs secreted by RAW cells. Gene expression of MGP was then quantified by quantitative real-time PCR and normalized to that of the housekeeping gene ß-actin. Data are expressed as the mean ± SEM of seven independent experiments performed in triplicate (*n* = 7). *p < 0.05 *vs*. 4 mM Pi, Mann-Whitney test. EV-CT: EVs derived from untreated macrophages, EV-LPS: EVs derived from LPS-EK-treated macrophages.

Oxidative stress and inflammation are two processes known to play an important role in vascular calcification. Our results show that EV-derived macrophages can induce both oxidative stress and inflammation. Thus, we next determined the effect of these EVs on Pi-induced calcification in smooth muscle cells. MOVAS cells were simultaneously treated with 4 mM Pi and macrophage-derived EVs for 14 days (Figure 12A) or macrophage-derived EVs for the last 7 days only (Figure 12B) of the induction of calcification. The intracellular calcium concentration in the smooth muscle cells was significantly higher after 14 days of treatment with Pi and EV-LPS than in cells treated with Pi and EV-CT ($ *p* < 0.05 *vs.* EV-CT, Figure 12A). Moreover, the intracellular calcium concentration was significantly higher after 7 days of treatment of Pi-treated smooth muscle cells with EV-LPS than Pi treatment alone (*p < 0.05 *vs.* 4 mM Pi, Figure 12B). These results suggest that EV-LPS can significantly induce calcification in MOVAS-1 cells. We can rule out that this effect was due to the presence of LPS-EK in EVs, as LPS-EK alone, with or without 4 mM Pi, had no effect on calcification (data not shown). We next measured the mRNA level of matrix gla protein (MGP), an inhibitor of vascular calcification. The level of MGP mRNA was significantly lower after 7 days of treatment of smooth muscle cells with 4 mM Pi and EV-LPS than in cells treated with Pi alone (*p < 0.05 *vs*. 4 mM Pi, Figure 13). The observed increase in calcification may therefore be partially due to a decrease in the level of calcification inhibitors, such as MGP.

**FIGURE 14:**
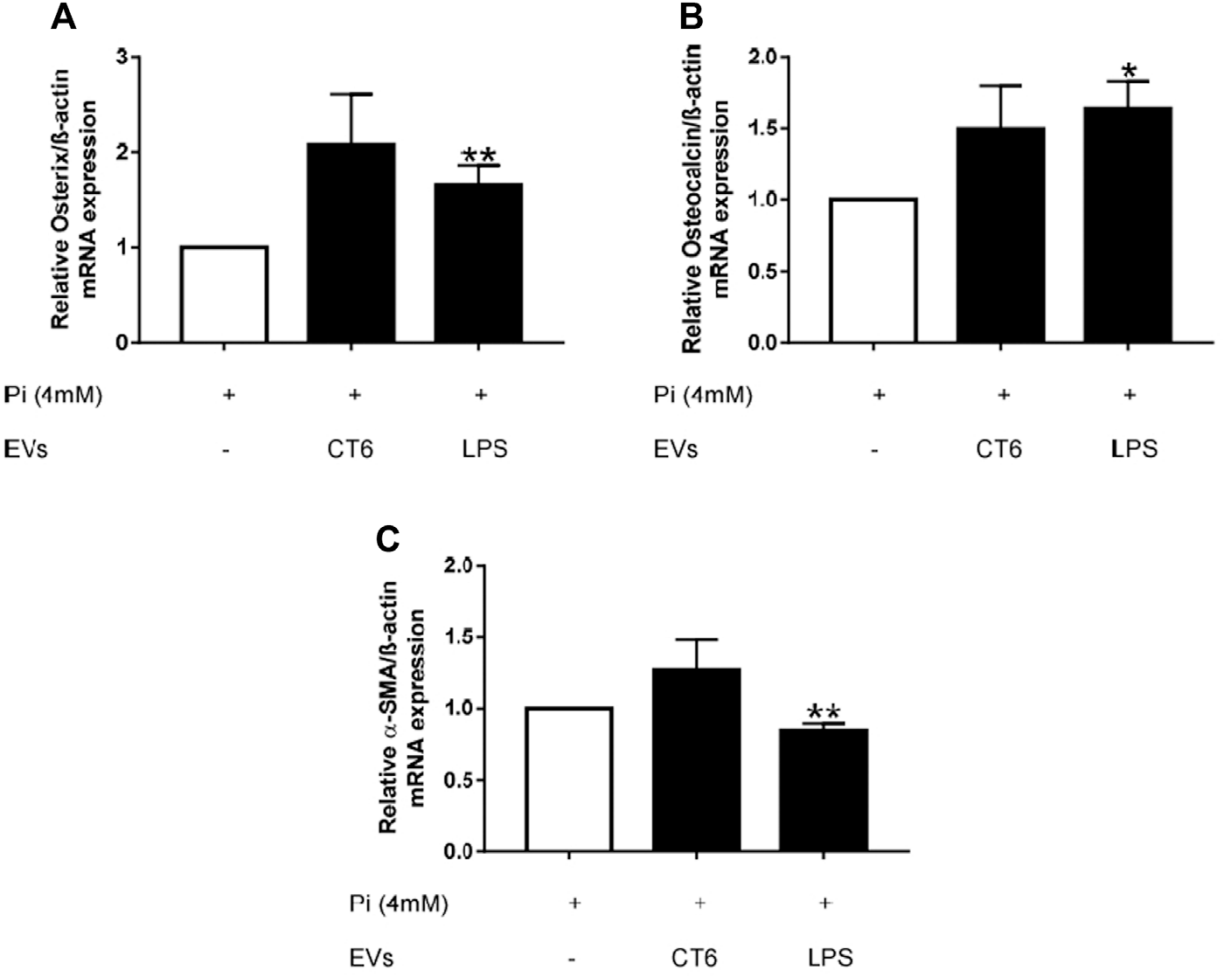
EV-LPS increase osteogenic marker gene expression and decrease contractile marker gene expression in smooth muscle cells. MOVAS-1 cells were incubated with 4 mM Pi, with or without RAW cell-derived EVs, for 7 days. Gene expression of **(A)** osterix, **(B)** osteocalcin, and **(C)** α-SMA was then quantified by quantitative real-time PCR and normalized to that of the housekeeping gene ß-actin. Data are expressed as the mean ± SEM of at least four independent experiments performed in triplicate (*n* = 4). *p < 0.05 *vs*. 4 mM Pi, **p < 0.01 *vs*. 4 mM Pi, Mann Whitney test. EV-CT: EVs derived from untreated macrophages, EV-LPS: EVs derived from LPS-EK-treated macrophages.

Smooth muscle cells can adopt a contractile, synthetic, or osteochondrogenic phenotype, depending on their environment (Durham et al., 2018). During vascular calcification, smooth muscle cells undergo an osteogenic switch to become osteoblast-like cells (Durham et al., 2018). We investigated whether this phenotypic change occurred in our experiments by measuring the mRNA levels of several osteogenic markers, such as osterix (Osx) and osteocalcin (OCN), as well as alpha-smooth muscle actin (α-SMA), a marker of the contractile phenotype. Osx and OCN mRNA levels were significantly higher in Pi-treated smooth muscle cells incubated with EV-LPS than Pi-treated smooth muscle cells, suggesting an osteogenic switch of the MOVAS-1 cells (*p < 0.05 *vs*. 4 mM Pi, **p < 0.01 *vs*. 4 mM Pi, Figures 14A,B). We also observed higher Osx and Ocn mRNA levels after treatment of Pi-treated smooth muscle cells with EV-CT, but without reaching significance. This result can be explained by the higher amount of EVs in the EV-CT preparation. At the same time, α-SMA mRNA levels were significantly lower than in Pi-treated smooth muscle cells (**p < 0.01 *vs*. 4 mM Pi, Figure 14C). Overall, these results show that EV-LPS induce an osteogenic switch of smooth muscle cells, which could, in turn, promote calcification.

## 4 Discussion

Vascular calcification (VC) is a complex process involving various molecular and cellular mechanisms, such as the VSMC osteogenic switch, loss of VC inhibitors, cell death, and dysregulation of Ca^2+^/Pi homeostasis, as well as matrix degradation and modification (Lee et al., 2020). Macrophages are among the main sources of inflammation and oxidative stress (Castaneda et al., 2017) and associated with arterial calcification (Agharazii et al., 2015). Indeed, Aikawa *et al.* showed co-localization of calcification and macrophages in atherosclerotic plaques of apolipoprotein E-deficient mice by image analysis, suggesting a prominent role of these cells and inflammation in calcification (Aikawa et al., 2007). In addition, a number of studies have shown macrophage-derived EVs to be mediators of VC (New et al., 2013; Chen et al., 2016; Li Y. et al., 2020; Kawakami et al., 2020). In this context, we hypothesized that macrophage-derived EVs secreted under pro-inflammatory and pro-oxidative conditions may increase VSMC calcification by propagating inflammation and oxidative stress. We tested our hypothesis by treating a murine VSMC cell line with EVs derived from LPS treated-murine macrophages. We then analyzed the effect of macrophage-derived EVs on the calcium content and levels of inflammatory, oxidative stress, and osteogenic markers in VSMCs. Moreover, we analyzed the protein content of EVs secreted by such activated macrophages.

We show that LPS-EK induces an inflammatory response in macrophages, as demonstrated by the significant increase in mRNA levels of pro-inflammatory cytokines (IL-6, IL-1ß, and TNF-α). These results are in accordance with those in the literature, as activation of TLR4 by LPS triggers activation of the MyD88-dependent pathway, inducing pro-inflammatory cytokine gene transcription. NLRP3, an activator protein of the inflammasome has also been shown to be significantly upregulated after LPS treatment (He et al., 2016). Our results show that LPS-EK induces pro-inflammatory M1 polarization of RAW cells, in accordance with the results of a study of Li *et al.* performed in the U937 monocyte cell line (Li et al., 2017). LPS-EK also induced oxidative stress, as shown by the significant increase in ROS production, as previously reported. In parallel, we showed a significant decrease in the expression of genes involved in the antioxidant system, such as Nrf2/Keap. Nrf2 is a transcriptional factor that induces the expression of antioxidant genes. Under basal conditions, Keap1 forms a complex with Nrf2 to induce its degradation by the proteasome. Under conditions of oxidative stress, modifications of the cysteine residues of Keap1 inhibit its interaction with Nrf2, allowing its nuclear translocation and protection of cells against oxidative stress. The observed decrease in Keap1 mRNA levels could be explained by the induction of protective mechanism activated by the cells to counteract ROS production. Surprisingly, despite an increase in ROS production, we showed a significant decrease in O_2_^•–^ production, which can be explained by both a decrease in Nox-2 levels and an increase in its degradation by SOD-2. This discrepancy can be partially explained by the fact that O_2_^•–^ can rapidly react with NO to form peroxynitrite (ONOO^−^), which mediates oxidative responses. Indeed, under LPS treatment, we observed a decrease in NO production, despite an increase in iNOS, an enzyme that produces NO.

As already shown (Liu et al., 2017; Bell et al., 2019), LPS-EK inhibited EV biogenesis by decreasing the expression of several markers of EV biogenesis, such as sphingomyelin phosphodiesterase 3 (SMPD3), phospho-1, and tissue nonspecific phosphatase alkaline phosphatase (TNAP). These results were confirmed by NTA analysis, which showed a reduced number of secreted EVs after LPS treatment. A similar result was reported by Bell *et al.* in AC16 human cardiomyocytes (Bell et al., 2019). In our cellular model, LPS-EK significantly decreased the expression of autophagic markers (Atg5, beclin1, ULK). Autophagy has already been shown to play an important role in EV biogenesis (Yang et al., 2021). This was confirmed by a significant increase in p62 expression after LPS treatment, as it has been shown that p62 protein accumulates upon inhibition of autophagy (Lamark et al., 2009). Interestingly, Liu et al. recently demonstrated a link between autophagy and macrophage polarization, showing that inhibition of autophagy induces the polarization of macrophages towards a pro-inflammatory M1 phenotype (Liu et al., 2015), a phenotype that we observed in our cells after treatment with LPS-EK. Moreover, Zhao *et al.* have shown that autophagy contributes to redox homeostasis, not only by clearing oxidized cellular components, but also by promoting antioxidant defenses *via* the p62/Keap1/Nrf2 pathway (Zhao et al., 2019). Therefore, a deficiency in autophagy, as observed in our cellular model, would decrease antioxidant defenses, which could also explain the observed increase in oxidative stress.

We investigated whether EVs secreted by LPS-treated cells play a role in VC by incubating VSMCs with EV-LPS. We show that EV-LPS induces inflammation and oxidative stress in VSMCs, with a significant increase in the expression of pro-inflammatory cytokines (IL6, IL1β, TNFα). Assessment of the inflammatory profile of the secreted EVs showed that EV-LPS are in fact enriched in these proinflammatory cytokines, consistent with the M1 polarization of RAW cells observed after treatment with LPS-EK. Cytokines are key players in cell-cell communication and play important roles in several biological processes, such as cell differentiation and inflammation. Various secretory pathways are responsible for their release, such as the classical ER/Golgi route and the unconventional pathway, also known as unconventional protein secretion (UPS). Recently, cytokines have been shown to be capable of reaching the extracellular milieu *via* a novel secretory pathway through EVs. Fitzgerald et al. thus showed that cytokine encapsulation into EVs is a general biological phenomenon observed *in vitro* and *in vivo* (Fitzgerald et al., 2018). Interestingly the profile of such encapsulated cytokines changes in response to various stimuli, suggesting that cytokine association with EVs is not specific to any particular cytokine, as all cytokines can be encapsulated. Tokarz et al. showed, for example, that the association of specific cytokines with EVs is strongly influenced by disease duration and treatment in diabetes (Tokarz et al., 2015). Such an association between cytokines and EVs appears to result from a specific physiological need, depending on whether the cytokines act near the Ev-secreting cells or at a distance. Indeed, Fitzgerald et al. (2018) showed that tissue explants, in which cells are in proximity with each other, secrete more soluble cytokine than cells in suspension, in which cytokines are more highly associated with EVs to allow their interaction with recipient cells at a distance. It is therefore possible that pro-inflammatory cytokines from M1 macrophages are carried by EVs secreted by these cells to induce a pro-inflammatory microenvironment for recipient cells, such as VSMCs. Moreover, several studies have shown that inflammatory cytokines have biological effects in VSMCs (Nilsson, 1993). For example, Barillari *et al.* showed that IL-1β, TNF-α, and IFN-γ released by activated immune cells enhance the expression of α5β1 integrin, a fibronectin receptor, leading to an increase in VSMC proliferation and migration, two mechanisms required for the formation of atherosclerotic lesions (Barillari et al., 2001). Our results also show that EV-LPS induce oxidative stress, as demonstrated by increased ROS production and decreased expression of antioxidant enzyme genes. However, EVs from non-treated cells (EV-CT) were also able to induce oxidative stress. Nevertheless, it is worthwhile noting that we treated cells with equivalent volumes of EV preparations. As LPS-treated cells secreted fewer EVs than control cells, it is likely that using the same volumes to treat VSMCs resulted in treating them with a lower number of EV-LPS than EV-CT, thus underestimating the effects of EV-LPS on VSMCs. These results confirm our hypothesis that EVs secreted under pro-inflammatory and pro-oxidative conditions are able to propagate inflammation and oxidative stress to surrounding cells, such as VSMCs. Finally, EV-LPS enhanced Pi-induced calcification of VSMCs by inducing the VSMC osteogenic switch and decreasing expression of the calcification inhibitor, MGP. These effects were intrinsic to EVs, as LPS alone had no effect on calcification. Moreover, in a similar manner, the study of Li *et al.* showed that the conditioned medium of LPS-treated macrophages induces an osteogenic switch of valve interstitial cells by increasing OPN, BMP-2, and ALP expression (Li et al., 2017). Furthermore, the aforementioned study showed that the increase in IL-6, IL-1β, and TNF-α in the conditioned medium was associated with an increase in the production of MMPs, which contribute to extracellular matrix degradation, remodeling, and valve calcification (Li et al., 2017).

Proteomic analysis highlighted the upregulation of three proteins in EV-LPS relative to EV-CT: cis-aconitate decarboxylase (CAD), plasminogen activator inhibitor-1 (PAI-1), and serum amyloid A-3 protein (Saa3). CAD, a mitochondrial enzyme encoded by immunoresponsive gene 1 (Irg1) and involved in itaconate production, is known to be upregulated in macrophages under pro-inflammatory conditions (Basler et al., 2006; Németh et al., 2016; Tallam et al., 2016; Song et al., 2020). Itaconate has been shown to be an immunoregulatory and anti-oxidant molecule (Lampropoulou et al., 2016; Mills et al., 2018; Li R. et al., 2020; Song et al., 2020). Itaconate has also been shown to promote IL-1β production and inflammatory apoptosis when administered at high doses to bone marrow-derived dendritic cells (Muri et al., 2020). In addition, the production of ROS can be mediated by Irg1 induction (Tan et al., 2016). In our study, EV-LPS were enriched in CAD. It is thus possible that this enzyme may induce the production of itaconate in VSMCs when transferred to the recipient cells via EVs, leading to inflammation and oxidative stress. PAI-1 is known to inhibit the action of plasminogen activators, such as tPA and uPA. First, several studies have already shown the upregulation of PAI-1 expression in LPS-treated cells (Wang et al., 2014; Ren et al., 2015) and an immunoregulatory role for PAI-1 through the TLR4 signaling pathway (Gupta et al., 2016). PAI-1 levels also increase under oxidative conditions (Vulin and Stanley, 2004). The higher amount of PAI-1 found in EV-LPS could be explained by macrophage activation. Numerous studies have shown PAI-1 to be associated with atherosclerotic lesions. Indeed, PAI-1 levels are high in atherosclerotic coronary arteries (Schneiderman et al., 1992; Lupu et al., 1993; Raghunath et al., 1995; Padró et al., 1997). PAI-1 expression was also found to be higher in CKD patients than healthy individuals (Ouyang et al., 2013). Furthermore, a number of studies have shown that PAI-1 is linked to VC. The upregulation of PAI-1 was, indeed, shown to be proportional to the calcium content in 65 calcified aortic valves (Kochtebane et al., 2014). Another study showed PAI-1 to positively correlate with vascular media thickness and calcification (Ouyang et al., 2013). PAI-1 transported by EV-LPS could thus participate in the aggravation of the VC process, as observed under our experimental conditions. Finally, Saa3, a member of apolipoproteins associated with high-density lipoprotein (HDL) in plasma, was only overexpressed in EV-LPS. As for CAD and PAI-1, Saa3 was upregulated after LPS treatment (Meek et al., 1992; Reigstad et al., 2009). Several studies have shown Saa proteins to have cytokine-like activity and to be able to activate several receptors, such as TLRs, and transcription factors, such as NF-κB (Ye and Sun, 2015). In addition, a number of studies have highlighted the role of Saa proteins in calcification (Tanaka et al., 2011; Ebert et al., 2015; Zhang et al., 2017). Saa proteins induce the production of pro-inflammatory cytokines and the osteogenic differentiation of mesenchymal stem cells *via* the TLR4 receptor (Ebert et al., 2015). Zhang *et al.* also showed that Saa proteins can induce the VSMC osteogenic switch through the p38 MAPK signaling pathway (Zhang et al., 2017). Furthermore, Saa proteins can increase calcium entry in human coronary artery smooth muscle cells (Tanaka et al., 2011). Thus, it is possible that Saa3 transported by EVs increases Pi entry into VSMCs and enhances the VSMC osteogenic switch.

In conclusion, we show a direct contribution of macrophages in the microcalcification process *via* EV secretion, an alternative pathway, in addition to the VSMC osteogenic switch. Indeed, EV-LPS, enriched for molecules involved in inflammation, oxidative stress, and VC mechanisms, were able to create an inflammatory microenvironment for surrounding cells, such as VSMCs, which in turn underwent an osteogenic switch, leading to increasing calcification. As atherosclerotic plaques containing microcalcifications are more susceptible to rupture and cardiovascular accidents, this study suggests that EVs could be used as non-invasive biomarkers to better stratify patients with a high risk of CV. Moreover, such EVs could therefore also be a therapeutic target to limit VC in patients.

## Data Availability

The data presented in the study are deposited in the PRIDE repository, accession number PXD029441(http://www.proteomexchange.org).

## References

[B1] Agharazii M. St-Louis R. Gautier-Bastien A. Ung R.-V. Mokas S. Larivière R. (2015). Inflammatory Cytokines and Reactive Oxygen Species as Mediators of Chronic Kidney Disease-Related Vascular Calcification. Am. J. Hypertens. 28, 746–755. 10.1093/ajh/hpu225 25430697

[B2] Aiello A. Giannessi F. Percario Z. A. Affabris E. (2020). An Emerging Interplay between Extracellular Vesicles and Cytokines. Cytokine Growth Factor. Rev. 51, 49–60. 10.1016/j.cytogfr.2019.12.003 31874738

[B3] Aikawa E. Nahrendorf M. Figueiredo J.-L. Swirski F. K. Shtatland T. Kohler R. H. (2007). Osteogenesis Associates with Inflammation in Early-Stage Atherosclerosis Evaluated by Molecular Imaging *In Vivo* . Circulation 116, 2841–2850. 10.1161/CIRCULATIONAHA.107.732867 18040026

[B4] Barillari G. Albonici L. Incerpi S. Bogetto L. Pistritto G. Volpi A. (2001). Inflammatory Cytokines Stimulate Vascular Smooth Muscle Cells Locomotion and Growth by Enhancing α5β1 Integrin Expression and Function. Atherosclerosis 154, 377–385. 10.1016/s0021-9150(00)00506-2 11166770

[B5] Basler T. Jeckstadt S. Valentin-Weigand P. Goethe R. (2006). Mycobacterium Paratuberculosis, Mycobacterium Smegmatis, and Lipopolysaccharide Induce Different Transcriptional and post-transcriptional Regulation of the IRG1 Gene in Murine Macrophages. J. Leukoc. Biol. 79, 628–638. 10.1189/jlb.0905520 16415166

[B6] Bell C. R. Jones L. B. Crenshaw B. J. Kumar S. Rowe G. C. Sims B. (2019). The Role of Lipopolysaccharide-Induced Extracellular Vesicles in Cardiac Cell Death. Biology 8, 69. 10.3390/biology8040069 PMC6955717 31547509

[B7] Bodega G. Alique M. Puebla L. Carracedo J. Ramírez R. M. (2019). Microvesicles: ROS Scavengers and ROS Producers. J. Extracellular Vesicles 8, 1626654. 10.1080/20013078.2019.1626654 31258880 PMC6586107

[B8] Castaneda O. A. Lee S.-C. Ho C.-T. Huang T.-C. (2017). Macrophages in Oxidative Stress and Models to Evaluate the Antioxidant Function of Dietary Natural Compounds. J. Food Drug Anal. 25, 111–118. 10.1016/j.jfda.2016.11.006 28911528 PMC9333431

[B9] Chen N. X. O’Neill K. D. Chen X. Moe S. M. (2008). Annexin-mediated Matrix Vesicle Calcification in Vascular Smooth Muscle Cells. J. Bone Mineral Res. 23, 1798–1805. 10.1359/jbmr.080604 PMC2685487 18597635

[B10] Chen Q. Bei J.-J. Liu C. Feng S.-B. Zhao W.-B. Zhou Z. (2016). HMGB1 Induces Secretion of Matrix Vesicles by Macrophages to Enhance Ectopic Mineralization. PLoS ONE 11, e0156686. 10.1371/journal.pone.0156686 27243975 PMC4887028

[B11] Cox J. Mann M. (2008). MaxQuant Enables High Peptide Identification Rates, Individualized p.p.b.-range Mass Accuracies and Proteome-wide Protein Quantification. Nat. Biotechnol. 26, 1367–1372. 10.1038/nbt.1511 19029910

[B12] Demer L. L. Tintut Y. (2008). Vascular Calcification. Circulation 117, 2938–2948. 10.1161/CIRCULATIONAHA.107.743161 18519861 PMC4431628

[B13] Drüeke T. B. Massy Z. A. (2011). Medial or Intimal Calcification in CKD-Does it Matter? Nat. Rev. Nephrol. 7, 250–251. 10.1038/nrneph.2011.41 21522194

[B14] Durham A. L. Speer M. Y. Scatena M. Giachelli C. M. Shanahan C. M. (2018). Role of Smooth Muscle Cells in Vascular Calcification: Implications in Atherosclerosis and Arterial Stiffness. Cardiovasc. Res. 114, 590–600. 10.1093/cvr/cvy010 29514202 PMC5852633

[B15] Ebert R. Benisch P. Krug M. Zeck S. Meißner-Weigl J. Steinert A. (2015). Acute Phase Serum Amyloid A Induces Proinflammatory Cytokines and Mineralization via Toll-like Receptor 4 in Mesenchymal Stem Cells. Stem Cel Res. 15, 231–239. 10.1016/j.scr.2015.06.008 26135899

[B16] Ellman G. L. Courtney K. D. Andres V. Featherstone R. M. (1961). A New and Rapid Colorimetric Determination of Acetylcholinesterase Activity. Biochem. Pharmacol. 7, 88–95. 10.1016/0006-2952(61)90145-9 13726518

[B17] Fitzgerald W. Freeman M. L. Lederman M. M. Vasilieva E. Romero R. Margolis L. (2018). A System of Cytokines Encapsulated in ExtraCellular Vesicles. Sci. Rep. 8, 8973. 10.1038/s41598-018-27190-x 29895824 PMC5997670

[B18] Fujii T. Mashimo M. Moriwaki Y. Misawa H. Ono S. Horiguchi K. (2017). Physiological Functions of the Cholinergic System in Immune Cells. J. Pharmacol. Sci. 134, 1–21. 10.1016/j.jphs.2017.05.002 28552584

[B19] Gupta K. K. Xu Z. Castellino F. J. Ploplis V. A. (2016). Plasminogen Activator Inhibitor-1 Stimulates Macrophage Activation through Toll-like Receptor-4. Biochem. Biophysical Res. Commun. 477, 503–508. 10.1016/j.bbrc.2016.06.065 27317488

[B20] He Y. Hara H. Núñez G. (2016). Mechanism and Regulation of NLRP3 Inflammasome Activation. Trends Biochem. Sci. 41, 1012–1021. 10.1016/j.tibs.2016.09.002 27669650 PMC5123939

[B21] Hénaut L. Candellier A. Boudot C. Grissi M. Mentaverri R. Choukroun G. (2019). New Insights into the Roles of Monocytes/Macrophages in Cardiovascular Calcification Associated with Chronic Kidney Disease. Toxins 11, 529. 10.3390/toxins11090529 PMC6784181 31547340

[B22] Hodroge A. Trécherel E. Cornu M. Darwiche W. Mansour A. Ait-Mohand K. (2017). Oligogalacturonic Acid Inhibits Vascular Calcification by Two Mechanisms. Atvb 37, 1391–1401. 10.1161/atvbaha.117.309513 28522698

[B23] Hu C.-T. Shao Y.-D. Liu Y.-Z. Xiao X. Cheng Z.-B. Qu S.-L. (2021). Oxidative Stress in Vascular Calcification. Clinica Chim. Acta 519, 101–110. 10.1016/j.cca.2021.04.012 33887264

[B24] Jaminon A. Reesink K. Kroon A. Schurgers L. (2019). The Role of Vascular Smooth Muscle Cells in Arterial Remodeling: Focus on Calcification-Related Processes. Ijms 20, 5694. 10.3390/ijms20225694 PMC6888164 31739395

[B25] Kawakami R. Katsuki S. Travers R. Romero D. C. Becker-Greene D. Passos L. S. A. (2020). S100A9-RAGE Axis Accelerates Formation of Macrophage-Mediated Extracellular Vesicle Microcalcification in Diabetes Mellitus. Atvb 40, 1838–1853. 10.1161/ATVBAHA.118.314087 PMC7377960 32460581

[B26] Kochtebane N. Alzahrani A. M. M. Bartegi A. (2014). Expression of uPA, tPA, and PAI-1 in Calcified Aortic Valves. Biochem. Res. Int. 2014, 1. 10.1155/2014/658643 PMC3947876 24693431

[B27] Komaba H. Fukagawa M. (2009). Fetuin-mineral Complex: a New Potential Biomarker for Vascular Calcification? Kidney Int. 75, 874–876. 10.1038/ki.2009.52 19367310

[B28] Lamark T. Kirkin V. Dikic I. Johansen T. (2009). NBR1 and P62 as Cargo Receptors for Selective Autophagy of Ubiquitinated Targets. Cell Cycle 8, 1986–1990. 10.4161/cc.8.13.8892 19502794

[B29] Lampropoulou V. Sergushichev A. Bambouskova M. Nair S. Vincent E. E. Loginicheva E. (2016). Itaconate Links Inhibition of Succinate Dehydrogenase with Macrophage Metabolic Remodeling and Regulation of Inflammation. Cel Metab. 24, 158–166. 10.1016/j.cmet.2016.06.004 PMC5108454 27374498

[B30] Lee S. J. Lee I.-K. Jeon J.-H. (2020). Vascular Calcification-New Insights into its Mechanism. Ijms 21, 2685. 10.3390/ijms21082685 PMC7216228 32294899

[B31] Li G. Qiao W. Zhang W. Li F. Shi J. Dong N. (2017). The Shift of Macrophages toward M1 Phenotype Promotes Aortic Valvular Calcification. J. Thorac. Cardiovasc. Surg. 153, 1318–1327. 10.1016/j.jtcvs.2017.01.052 28283241

[B32] Li R. Zhang P. Wang Y. Tao K. (2020a). Itaconate: A Metabolite Regulates Inflammation Response and Oxidative Stress. Oxidative Med. Cell Longevity 2020, 1. 10.1155/2020/5404780 PMC7382747 32724492

[B33] Li Y. Sun Z. Zhang L. Yan J. Shao C. Jing L. (2020b). Role of Macrophages in the Progression and Regression of Vascular Calcification. Front. Pharmacol. 11. 10.3389/fphar.2020.00661 PMC7227444 32457633

[B34] Liu F. Li X. Yue H. Ji J. You M. Ding L. (2017). TLR-induced SMPD3 Defects Enhance Inflammatory Response of B Cell and Macrophage in the Pathogenesis of SLE. Scand. J. Immunol. 86, 377–388. 10.1111/sji.12611 28889482

[B35] Liu K. Zhao E. Ilyas G. Lalazar G. Lin Y. Haseeb M. (2015). Impaired Macrophage Autophagy Increases the Immune Response in Obese Mice by Promoting Proinflammatory Macrophage Polarization. Autophagy 11, 271–284. 10.1080/15548627.2015.1009787 25650776 PMC4502775

[B36] Lupu F. Bergonzelli G. E. Heim D. A. Cousin E. Genton C. Y. Bachmann F. (1993). Localization and Production of Plasminogen Activator Inhibitor-1 in Human Healthy and Atherosclerotic Arteries. Arterioscler Thromb. 13, 1090–1100. 10.1161/01.atv.13.7.1090 7686395

[B37] Mansour A. Darwiche W. Yaker L. Da Nascimento S. Gomila C. Rossi C. (2020). GFOGER Peptide Modifies the Protein Content of Extracellular Vesicles and Inhibits Vascular Calcification. Front. Cel Dev. Biol. 8, 589761. 10.3389/fcell.2020.589761 PMC7734313 33330469

[B38] Mastronarde D. N. (2005). Automated Electron Microscope Tomography Using Robust Prediction of Specimen Movements. J. Struct. Biol. 152, 36–51. 10.1016/j.jsb.2005.07.007 16182563

[B39] Meek R. L. Eriksen N. Benditt E. P. (1992). Murine Serum Amyloid A3 Is a High Density Apolipoprotein and Is Secreted by Macrophages. Proc. Natl. Acad. Sci. 89, 7949–7952. 10.1073/pnas.89.17.7949 1518819 PMC49832

[B40] Mills E. L. Ryan D. G. Prag H. A. Dikovskaya D. Menon D. Zaslona Z. (2018). Itaconate Is an Anti-inflammatory Metabolite that Activates Nrf2 via Alkylation of KEAP1. Nature 556, 113–117. 10.1038/nature25986 29590092 PMC6047741

[B41] Moe S. M. Chen N. X. (2005). Inflammation and Vascular Calcification. Blood Purif. 23, 64–71. 10.1159/000082013 15627739

[B42] Muri J. Wolleb H. Broz P. Carreira E. M. Kopf M. (2020). Electrophilic Nrf2 Activators and Itaconate Inhibit Inflammation at Low Dose and Promote IL-1β Production and Inflammatory Apoptosis at High Dose. Redox Biol. 36, 101647. 10.1016/j.redox.2020.101647 32863237 PMC7387846

[B43] Németh B. Doczi J. Csete D. Kacso G. Ravasz D. Adams D. (2016). Abolition of Mitochondrial Substrate‐level Phosphorylation by Itaconic Acid Produced by LPS‐induced Irg1 Expression in Cells of Murine Macrophage Lineage. FASEB j. 30, 286–300. 10.1096/fj.15-279398 26358042

[B44] New S. E. P. Goettsch C. Aikawa M. Marchini J. F. Shibasaki M. Yabusaki K. (2013). Macrophage-Derived Matrix Vesicles. Circ. Res. 113, 72–77. 10.1161/CIRCRESAHA.113.301036 23616621 PMC3703850

[B45] Nilsson J. (1993). Cytokines and Smooth Muscle Cells in Atherosclerosis. Cardiovasc. Res. 27, 1184–1190. 10.1093/cvr/27.7.1184 8252576

[B46] Ouyang L. Peng Y. Wu G. Xu X. He Z. (2013). Efffect of Plasminogen Activator Inhibitor-1 and Endothelin-1 on the Atherosclerosis in the Maintenance Hemodialysis Patients. Zhong Nan Da Xue Xue Bao Yi Xue Ban 38, 458–467. 10.3969/j.issn.1672-7347.2013.05.004 23719522

[B47] Padró T. Steins M. Li C.-X. Mesters R. M. Hammel D. Scheld H. H. (1997). Comparative Analysis of Plasminogen Activator Inhibitor-1 Expression in Different Types of Atherosclerotic Lesions in Coronary Arteries from Human Heart Explants. Cardiovasc. Res. 36, 28–36. 10.1016/S0008-6363(97)00144-2 9415269

[B48] Perez-Riverol Y. Csordas A. Bai J. Bernal-Llinares M. Hewapathirana S. Kundu D. J. (2019). The PRIDE Database and Related Tools and Resources in 2019: Improving Support for Quantification Data. Nucleic Acids Res. 47 (D1), D442–D450. 10.1093/nar/gky1106 30395289 PMC6323896

[B49] Peterson G. L. (1977). A simplification of the protein assay method of Lowry et al. which is more generally applicable. Anal. Biochem. 83, 346–356. 10.1016/0003-2697(77)90043-4 603028

[B50] Pi J. Li T. Liu J. Su X. Wang R. Yang F. (2014). Detection of Lipopolysaccharide Induced Inflammatory Responses in RAW264.7 Macrophages Using Atomic Force Microscope. Micron 65, 1–9. 10.1016/j.micron.2014.03.012 25041825

[B51] Qin Z. Liao R. Xiong Y. Jiang L. Li J. Wang L. (2021). A Narrative Review of Exosomes in Vascular Calcification. Ann. Transl. Med. 9, 579. 10.21037/atm-20-7355 33987277 PMC8105793

[B52] Raghunath P. N. Tomaszewski J. E. Brady S. T. Caron R. J. Okada S. S. Barnathan E. S. (1995). Plasminogen Activator System in Human Coronary Atherosclerosis. Atvb 15, 1432–1443. 10.1161/01.atv.15.9.1432 7670959

[B53] Raschke W. C. Baird S. Ralph P. Nakoinz I. (1978). Functional Macrophage Cell Lines Transformed by Abelson Leukemia Virus. Cell 15, 261–267. 10.1016/0092-8674(78)90101-0 212198

[B54] Ray Sarkar B. C. Chauhan U. P. S. (1967). A New Method for Determining Micro Quantities of Calcium in Biological Materials. Anal. Biochem. 20, 155–166. 10.1016/0003-2697(67)90273-4 6071917

[B55] Reigstad C. S. Lundén G. Ö. Felin J. Bäckhed F. (2009). Regulation of Serum Amyloid A3 (SAA3) in Mouse Colonic Epithelium and Adipose Tissue by the Intestinal Microbiota. PLOS ONE 4, e5842. 10.1371/journal.pone.0005842 19513118 PMC2688757

[B56] Ren W. Wang Z. Hua F. Zhu L. (2015). Plasminogen Activator Inhibitor-1 Regulates LPS-Induced TLR4/MD-2 Pathway Activation and Inflammation in Alveolar Macrophages. Inflammation 38, 384–393. 10.1007/s10753-014-0042-8 25342286

[B57] Schneiderman J. Sawdey M. S. Keeton M. R. Bordin G. M. Bernstein E. F. Dilley R. B. (1992). Increased Type 1 Plasminogen Activator Inhibitor Gene Expression in Atherosclerotic Human Arteries. Proc. Natl. Acad. Sci. 89, 6998–7002. 10.1073/pnas.89.15.6998 1495992 PMC49632

[B58] Song H. Xu T. Feng X. Lai Y. Yang Y. Zheng H. (2020). Itaconate Prevents Abdominal Aortic Aneurysm Formation through Inhibiting Inflammation via Activation of Nrf2. EBioMedicine 57, 102832. 10.1016/j.ebiom.2020.102832 32574955 PMC7322255

[B59] Tallam A. Perumal T. M. Antony P. M. Jäger C. Fritz J. V. Vallar L. (2016). Gene Regulatory Network Inference of Immunoresponsive Gene 1 (IRG1) Identifies Interferon Regulatory Factor 1 (IRF1) as its Transcriptional Regulator in Mammalian Macrophages. PLOS ONE 11, e0149050. 10.1371/journal.pone.0149050 26872335 PMC4752512

[B60] Tan H.-Y. Wang N. Li S. Hong M. Wang X. Feng Y. (2016). The Reactive Oxygen Species in Macrophage Polarization: Reflecting its Dual Role in Progression and Treatment of Human Diseases. Oxidative Med. Cell Longevity 2016, 1. 10.1155/2016/2795090 PMC4837277 27143992

[B61] Tanaka T. Ikeda K. Yamamoto Y. Iida H. Kikuchi H. Morita T. (2011). Effects of Serum Amyloid a and Lysophosphatidylcholine on Intracellular Calcium Concentration in Human Coronary Artery Smooth Muscle Cells. Int. Heart J. 52, 185–193. 10.1536/ihj.52.185 21646743

[B62] Théry C. Witwer K. W. Aikawa E. Alcaraz M. J. Anderson J. D. Andriantsitohaina R. (2018). Minimal Information for Studies of Extracellular Vesicles 2018 (MISEV2018): a Position Statement of the International Society for Extracellular Vesicles and Update of the MISEV2014 Guidelines. J. Extracell. Vesicles 7, 1535750. 10.1080/20013078.2018.1535750 30637094 PMC6322352

[B63] Tokarz A. Szuścik I. Kuśnierz-Cabala B. Kapusta M. Konkolewska M. Żurakowski A. (2015). Extracellular Vesicles Participate in the Transport of Cytokines and Angiogenic Factors in Diabetic Patients with Ocular Complications. Folia Med. Cracov 55, 35–48. 26867118

[B64] Tóth A. Balogh E. Jeney V. (2020). Regulation of Vascular Calcification by Reactive Oxygen Species. Antioxidants 9, 963. 10.3390/antiox9100963 PMC7599480 33049989

[B65] Tyanova S. Temu T. Sinitcyn P. Carlson A. Hein M. Y. Geiger T. (2016). The Perseus Computational Platform for Comprehensive Analysis of (Prote)omics Data. Nat. Methods 13, 731–740. 10.1038/nmeth.3901 27348712

[B66] van Niel G. D'Angelo G. Raposo G. (2018). Shedding Light on the Cell Biology of Extracellular Vesicles. Nat. Rev. Mol. Cel Biol. 19, 213–228. 10.1038/nrm.2017.125 29339798

[B67] Voelkl J. Lang F. Eckardt K.-U. Amann K. Kuro-O M. Pasch A. (2019). Signaling Pathways Involved in Vascular Smooth Muscle Cell Calcification during Hyperphosphatemia. Cell. Mol. Life Sci. 76, 2077–2091. 10.1007/s00018-019-03054-z 30887097 PMC6502780

[B68] Vulin A. I. Stanley F. M. (2004). Oxidative Stress Activates the Plasminogen Activator Inhibitor Type 1 (PAI-1) Promoter through an AP-1 Response Element and Cooperates with Insulin for Additive Effects on PAI-1 Transcription. J. Biol. Chem. 279, 25172–25178. 10.1074/jbc.M403184200 15069077

[B69] Wang Z.-H. Ren W.-Y. Zhu L. Hu L.-J. (2014). Plasminogen Activator Inhibitor-1 Regulates LPS Induced Inflammation in Rat Macrophages through Autophagy Activation. Scientific World J. 2014, 1. 10.1155/2014/189168 PMC4122156 25133205

[B70] Woith E. Fuhrmann G. Melzig M. F. (2019). Extracellular Vesicles-Connecting Kingdoms. Ijms 20, 5695. 10.3390/ijms20225695 PMC6888613 31739393

[B71] Yaker L. Kamel S. Ausseil J. Boullier A. (2020). Effects of Chronic Kidney Disease and Uremic Toxins on Extracellular Vesicle Biology. Toxins 12, 811. 10.3390/toxins12120811 33371311 PMC7767379

[B72] Yang X. Song X. Li Z. Liu N. Yan Y. Liu B. (202110562). Crosstalk between Extracellular Vesicles and Autophagy in Cardiovascular Pathophysiology. Pharmacol. Res. 172, 105628. 10.1016/j.phrs.2021.105628 33887437

[B73] Ye R. D. Sun L. (2015). Emerging Functions of Serum Amyloid A in Inflammation. J. Leukoc. Biol. 98, 923–929. 10.1189/jlb.3VMR0315-080R 26130702 PMC6608020

[B74] Zhang X. Chen J. Wang S. (2017). Serum Amyloid A Induces a Vascular Smooth Muscle Cell Phenotype Switch through the P38 MAPK Signaling Pathway. Biomed. Res. Int. 2017, 1. 10.1155/2017/4941379 PMC5469989 28642873

[B75] Zhao L. Li H. Wang Y. Zheng A. Cao L. Liu J. (2019). Autophagy Deficiency Leads to Impaired Antioxidant Defense via P62-Foxo1/3 Axis. Oxidative Med. Cell Longevity 2019, 1–15. 10.1155/2019/2526314 PMC6935825 31949875

