# Supplementary material for: Extracellular Vesicles From LPS-Treated Macrophages Aggravate Smooth Muscle Cell Calcification by Propagating Inflammation and Oxidative Stress
Source: Front Cell Dev Biol. 2022 Mar 9;10:823450. doi: 10.3389/fcell.2022.823450 (PMC8959646; doi:10.3389/fcell.2022.823450)
Supplement: Supplementary file 1 [file DataSheet2.PDF]

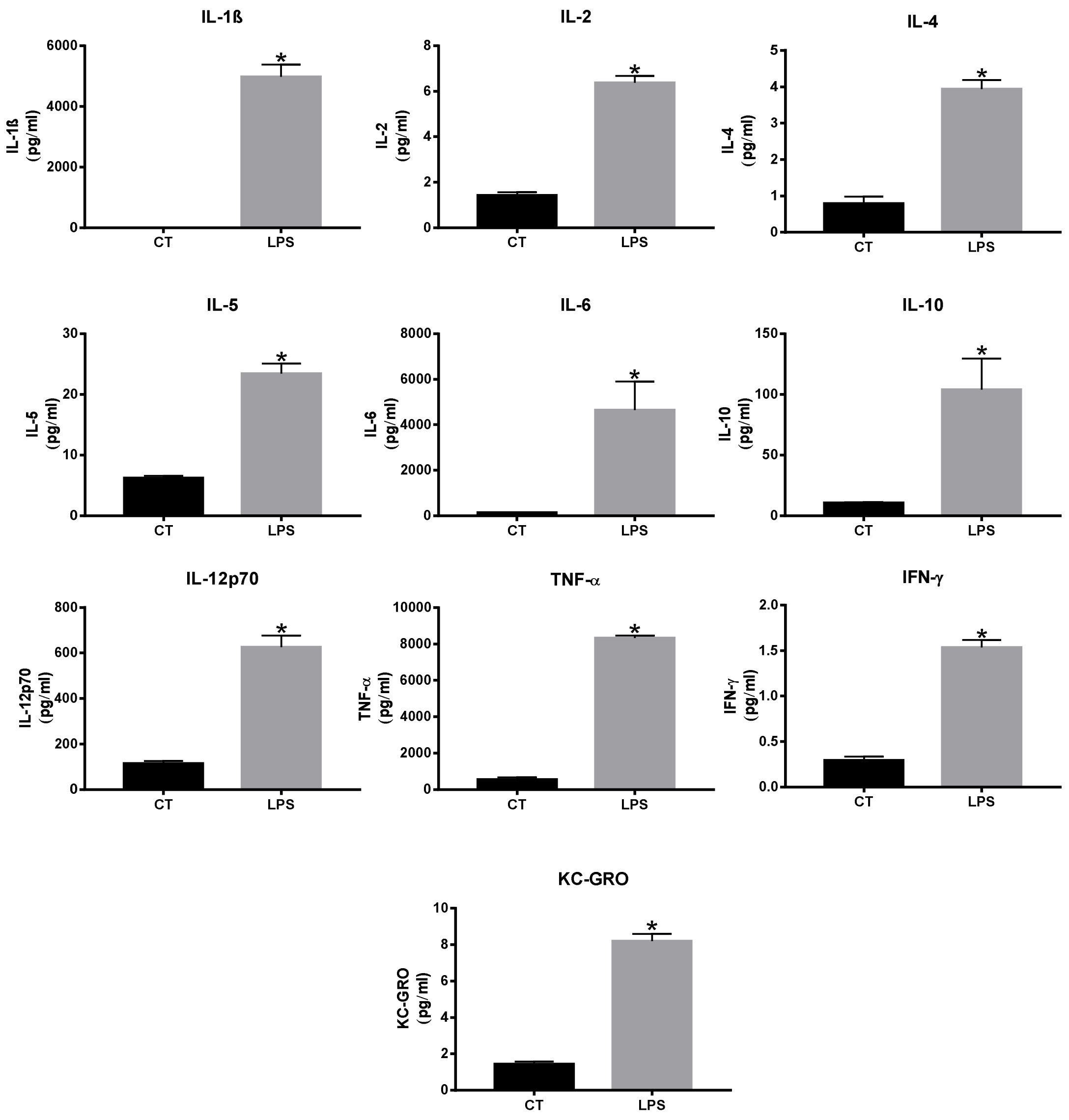

Supplemental Figure S2. LPS-EK increases the concentration of 10 proinflammatory cytokines in macrophages. RAW cells were treated with (LPS-EK) or without (CT) 1µg/ml lipopolysaccharide-EK for 6 h (LPS). Proinflammatory cytokine protein levels (IL-1ß, IL-2, IL-4, IL-5, IL-6, IL-10, IL-12p70, TNF-α, IFN-γ, and KC-GRO) were measured in macrophages using an MSD multiplex immunoassay. Data are expressed as the mean ± SEM of four independent experiments performed in duplicate (n = 4). *$p < 0.05$ *vs*. CT, Mann-Whitney test.
