# Supplementary material for: Extracellular Vesicles From LPS-Treated Macrophages Aggravate Smooth Muscle Cell Calcification by Propagating Inflammation and Oxidative Stress
Source: Front Cell Dev Biol. 2022 Mar 9;10:823450. doi: 10.3389/fcell.2022.823450 (PMC8959646; doi:10.3389/fcell.2022.823450)
Supplement: Supplementary file 2 [file DataSheet4.PDF]

Supplementary Material

Supplementary Table 1. Primers used for quantitative real-time PCR.

Gene	Forward Primer	Reverse Primer
IL-6	5'- AGCCAGAGTCCTTCAGAGAGATAC -3'	5'- AATTGGATGGTCTTGGTCCTTAGC -3'
IL-1β	5'- ACCTTCCAGGATGAGGACATGA -3'	5'- CTAATGGGAACGTCACACACCA -3'
TNF-α	5'- ATGAGAAGTTCCCAAATGGCC -3'	5'- CCACTTGGTGGTTTGCTACGA -3'
MIP-2	5'- CTGGGGAGAGGGTGAGTTG -3'	5'- GCTGTTCTACTCTCCTCGGTG -3'
NLRP3	5'- ATTACCCGCCCGAGAAAGG -3'	5'- CATGAGTGTGGCTAGATCCAAG -3'
SOD-1	5'- GTGCAGGGAACCATCCACTT -3'	5'- GTCCTGACAACACAACTGGTTCA -3'
SOD-2	5'- GCGGCCTACGTGAACAATCT -3'	5'- ATATGTCCCCCACCATTGAACT -3'
iNOS	5'- CCCTTCAATGGTTGGTACATGG -3'	5'- ACATTGATCTCCGTGACAGCC -3'
Nox-2	5'- CTGCTCTCCTTTCTCAGGGGT -3'	5'- GTGTGCAGTGCTATCATCCAA -3'
Nrf2	5'- TTCTTTCAGCAGCATCCTCTCCAC -3'	5'- ACAGCCTTCAATAGTCCCGTCCAG -3'
Keap1	5'- ACGTCCTCGGAGGCTATGAT -3'	5'- TTTGCTTCCGACAGGGTTCC -3'
SMPD3	5'- AGAAACCCGGTCCTCGTACT -3'	5'- CCTGACCAGTGCCATTCTTT -3'
Phospho-1	5'- TTCTCATTTCGGATGCCA -3'	5'- TGAGGATGCGGCGGAAT -3'
TNAP	5'- CTGCCACTGCCTACTTGTGT -3'	5'- GATGGATGTGACCTCATTGC -3'
ULK1	5'- CCAGGCAGACATTGAGAACA -3'	5'- GTTGGCAGCAGGTAGTCAGG -3'
Atg5	5'- GCCGAACCCTTTGCTCAATG -3'	5'- TGGTCACCTTAGGAAATACCCAC -3'
LC3a	5'- AGCTTCGCCGACCGCTGTAAG -3'	5'- CTTCTCCTGTTCATAGATGTCAGC -3'
LC3b	5'- CGGAGCTTTGAACAAAGAGTG -3'	5'- TCTCTCACTCTCGTACACTTC -3'
Beclin-1	5'- TTTTCTGGACTGTGTGCAGC -3'	5'- GCTTTTGTCCACTGCTCCTC -3'
p62	5'- GCTCAGGAGGAGACGATGAC -3'	5'- AGAAACCCAAGGACAGCATC -3'
Osterix	5'- AGCGACCACTTGAGCAAACAT -3'	5'- GCGGCTGATTGGCTTCTTCT -3'
Osteocalcin	5'- GACCGCCTACAAACGCATCT -3'	5'- GGGCAGCACAGGTCCTAAATAGT -3'
α-SMA	5'- TGTGCTGGACTCTGGAGATG -3'	5'- GAAGGAATAGCCACGCTCAG -3'
MGP	5'- ATGAAGAGCCTGCTCCCTCT -3'	5'- ATATTTGGCTCCTCGGCGCT -3'
β-actin	5'- AGAGGGAAATCGTGCGTGAC -3'	5'- CAATAGTGATGACCTGGCCGT -3'

Supplementary Table 2. Detection of extracellular vesicle (EV) proteins and proteins associated with EV pathophysiology by mass spectrometry.

EV Protein Families	Detected Proteins
**Tetraspanins**	CD9, CD81, CD63
**ESCRT components**	Alix, Tsg101, Vps4a, Vps4b, Vps25, Vps28, Vps36, Vps37b, Chmp2a, Chmp2b, Chmp3, Chmp4b, Chmp6, Flotillin-1, Flotillin-2, and Syntenin-1
**Rab GTPases**	Rab5a, Rab5b, Rab5c, Rab7a, Rab9a, Rab27a, and Rab35
**Various Proteins associated with EV pathophysiology**	• Integrins ß1 and ß5<br>• Annexins A2, 5, and 6<br>• Inorganic Pyrophosphatase 2<br>• Kinases (Tyrosine Kinase and MAPK)<br>• Scramblases 1 and 3<br>• Phospholipase D4<br>• Glutathione-S-Transferase<br>• Apoptosis inducing factors (BAX)<br>• Cytokine receptor-like factor 3
