# Supplementary material for: Extracellular Vesicles From LPS-Treated Macrophages Aggravate Smooth Muscle Cell Calcification by Propagating Inflammation and Oxidative Stress
Source: Front Cell Dev Biol. 2022 Mar 9;10:823450. doi: 10.3389/fcell.2022.823450 (PMC8959646; doi:10.3389/fcell.2022.823450)
Supplement: Supplementary file 3 [file DataSheet3.PDF]

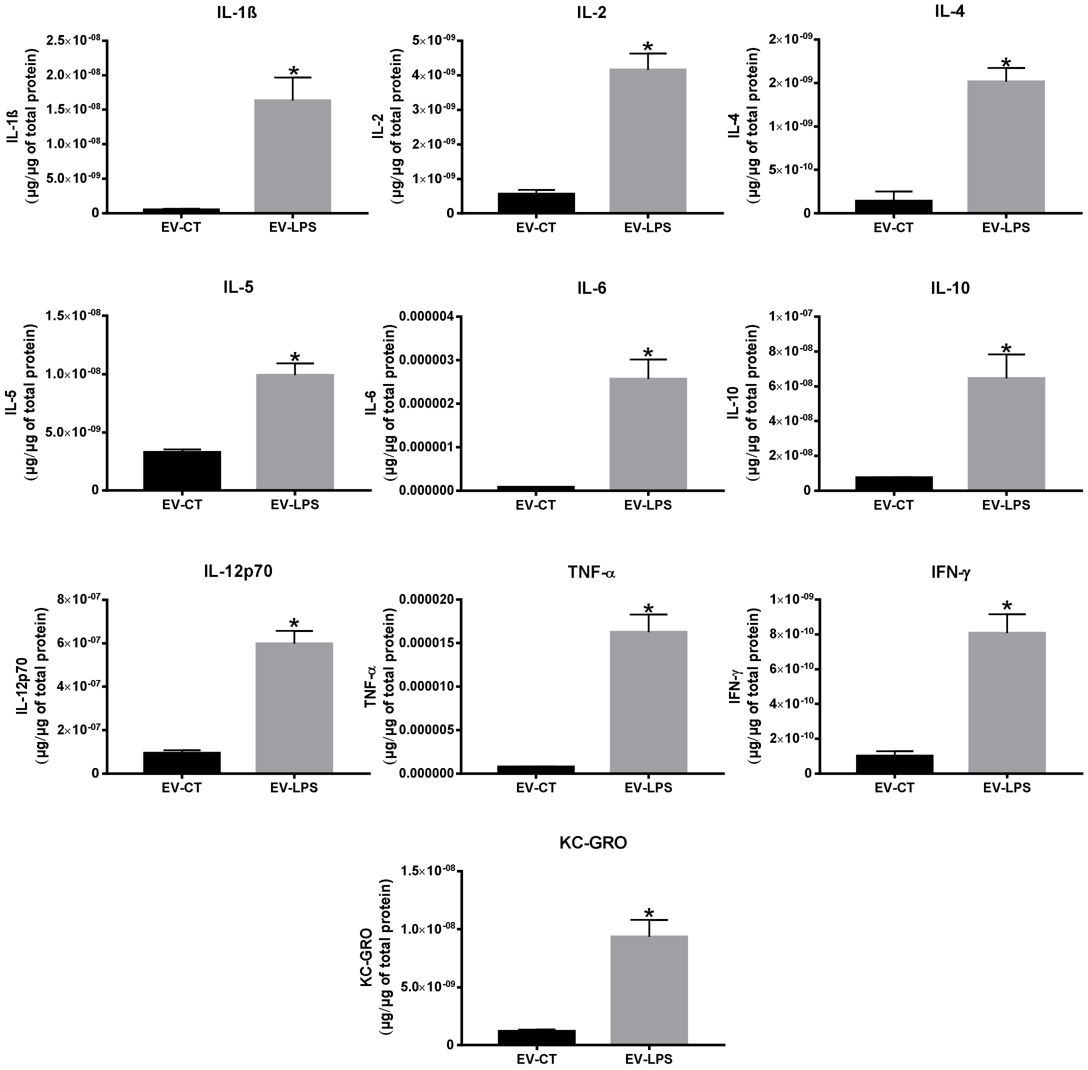

Supplemental figure S3. LPS-EK increases the concentration of 10 proinflammatory cytokines in macrophage-derived EVs. EVs were isolated from the culture media of RAW cells incubated with (EV-LPS) or without (EV-CT) lipopolysaccharide-EK (LPS-EK). Proinflammatory cytokine protein levels (IL-1ß, IL-2, IL-4, IL-5, IL-6, IL-10, IL-12p70, TNF-α, IFN-γ, and KC-GRO) were measured in macrophage-derived EVs using an MSD multiplex immunoassay. Data are expressed as the mean ± SEM of four independent experiments performed in duplicate (n = 4). *$p < 0.05$ *vs*. CT, Mann-Whitney test.
