# Supplementary material for: Extracellular Vesicles From LPS-Treated Macrophages Aggravate Smooth Muscle Cell Calcification by Propagating Inflammation and Oxidative Stress
Source: Front Cell Dev Biol. 2022 Mar 9;10:823450. doi: 10.3389/fcell.2022.823450 (PMC8959646; doi:10.3389/fcell.2022.823450)
Supplement: Supplementary file 4 [file DataSheet1.PDF]

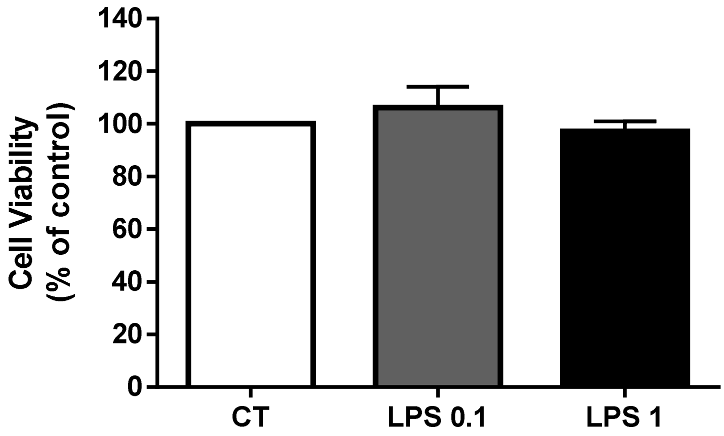

Supplemental Figure S1. Effect of lipopolysaccharide-EK (LPS-EK) on RAW cell viability. RAW cells were incubated with LPS-EK for 6 h. Cell viability was measured using the WST-1 assay. The viability of untreated control cells (CT) was defined as 100%. Data are expressed as the mean ± SEM of three independent experiments performed in triplicate (n = 3). LPS 0.1: 0.1 µg/ml LPS-EK, LPS 1: 1 µg/ml LPS-EK
